# Construction of high coverage whole-genome sequencing libraries from single colon crypts without DNA extraction or whole-genome amplification

**DOI:** 10.1186/s13104-023-06333-y

**Published:** 2023-04-27

**Authors:** Zarko Manojlovic, Jordan Wlodarczyk, Cindy Okitsu, Yuxin Jin, David Van Den Berg, Michael R. Lieber, Chih-Lin Hsieh

**Affiliations:** 1 Department of Urology, University of Southern California, 1441 Eastlake Ave., NTT5420, Los Angeles, CA USA; 2 Department of Surgery, University of Southern California, Los Angeles, CA USA; 3 Department of Pathology, University of Southern California, Los Angeles, CA USA; 4 Department of Translational Genomics, Keck School of Medicine of USC, Los Angeles, CA USA; 5 Department of Population and Public Health Sciences, University of Southern California, Los Angeles, CA USA

**Keywords:** Next generation sequencing, High coverage whole-genome sequencing, Sequencing library construction without DNA extraction

## Abstract

**Objective:**

Comprehensive and reliable genome-wide variant analysis of a small number of cells has been challenging due to genome coverage bias, PCR over-cycling, and the requirement of expensive technologies. To comprehensively identify genome alterations in single colon crypts that reflect genome heterogeneity of stem cells, we developed a method to construct whole-genome sequencing libraries from single colon crypts without DNA extraction, whole-genome amplification, or increased PCR enrichment cycles.

**Results:**

We present post-alignment statistics of 81 single-crypts (each contains four- to eight-fold less DNA than the requirement of conventional methods) and 16 bulk-tissue libraries to demonstrate the consistent success in obtaining reliable coverage, both in depth (≥ 30X) and breadth (≥ 92% of the genome covered at ≥ 10X depth), of the human genome. These single-crypt libraries are of comparable quality as libraries generated with the conventional method using high quality and quantities of purified DNA. Conceivably, our method can be applied to small biopsy samples from many tissues and can be combined with single cell targeted sequencing to comprehensively profile cancer genomes and their evolution. The broad potential application of this method offers expanded possibilities in cost-effectively examining genome heterogeneity in small numbers of cells at high resolution.

**Supplementary Information:**

The online version contains supplementary material available at 10.1186/s13104-023-06333-y.

## Introduction

Whole-genome sequencing (WGS) library preparation generally requires > 50 ng of purified human DNA input, equivalent to 8,333 diploid cells (not considering losses during DNA extraction), to achieve 30X average depth and > 90% breadth coverage of the genome for a comprehensive DNA variant analysis. Using tissue culture and whole-genome amplification (WGA) with limited starting material can introduce mutations and genome coverage bias. These problems persist despite the recent development of single-cell sequencing technology; therefore, comprehensive variant analysis without sequencing multiple cells cannot be achieved [1–4].

Our goal is to use the colon crypt, which consists of 1,000 to 2,000 cells all derived from a single stem cell [5–8], as a model system to identify mutations occurring in the single stem cells to understand genome heterogeneity. To achieve this goal, we modified the conventional WGS library preparation steps to generate high-quality sequencing libraries reaching a balanced ≥ 30X depth post-alignment coverage consistently from 81 single human colon crypts without WGA or DNA purification. This method will empower researchers to reliably generate streamlined high-quality libraries from tissues containing small numbers of cells.

## Materials and methods

### Tissue collection and crypt isolation

A small piece of colon is collected from individuals who have undergone surgery to remove part of the colon under the standard of care at either Keck Hospital of USC or Children’s Hospital Los Angeles through the Norris Comprehensive Cancer Center Translational Pathology Core.

A 5 mm x 5 mm colon specimen is cut into smaller pieces, washed with 10 ml 1X Phosphate Buffered Saline with 9 mM EDTA (1XPBS/EDTA) three times, and incubated in 1XPBS/EDTA for 20 min at room temperature. The liquid is decanted after incubation and 2 ml of 1XPBS (without EDTA) is added. After 10-second vortexing at high speed, 20 to 30 individual crypts are identified and transferred under an inverted microscope into separate low-binding microfuge tubes. The presence of one single colon crypt in each tube is confirmed under the microscope before storing at -80^o^C. The remaining crypts in the suspension are spun down for bulk DNA extraction using the phenol/chloroform extraction and ethanol precipitation method.

### Whole-genome sequencing library construction

**Fig. 1:**
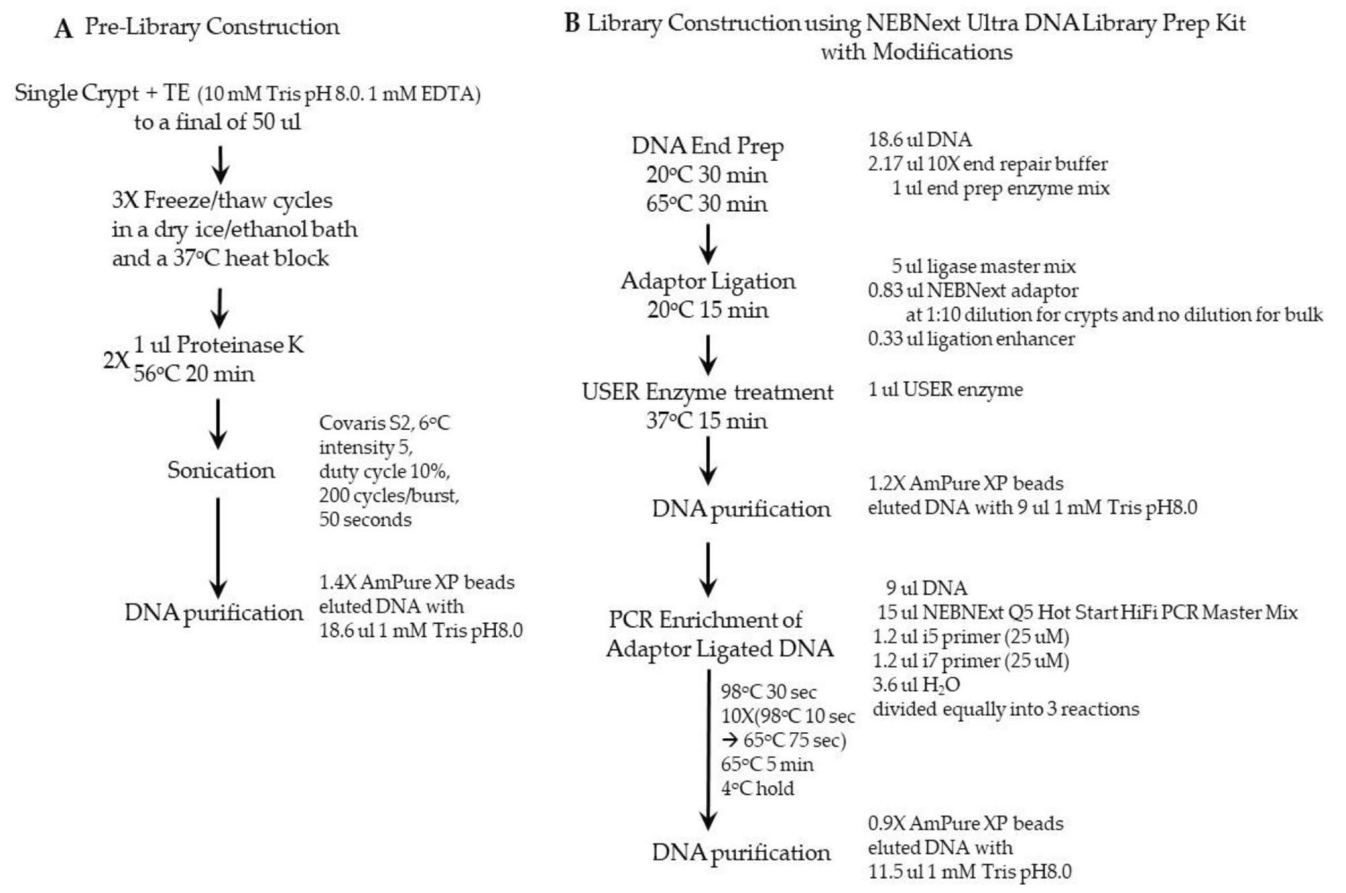
Whole-genome sequencing library construction from single colon crypts without DNA extraction. (A) Preparation and fragmentation of single colon crypt for library construction. (B) Workflow of library construction

The detailed workflow of single colon crypt sequencing library construction is illustrated in Fig. 1. The entire workflow through the enrichment step must be carried out without delay between each step to minimize DNA loss, and low-binding tubes are preferred. Specifically, reagents are pre-aliquoted or ready to be aliquoted just prior to each step, and no more than eight libraries are processed in each session. After freeze-and-thaw cycles, each colon crypt sample is treated with proteinase K, transferred to a Covaris microtube, and immediately sonicated and transferred into a fresh microtube containing 70 ul of pre-aliquoted AmPure XP beads (Backman Coulter). Throughout the experiment, the bead purification is done according to the manufacturer’s instruction, and the DNA is eluted with 1 mM Tris pH 8.0 preheated to 55^o^C.

The NEBNext Ultra DNA Library Prep Kit (NEB #E7370) is used according to the manufacturer’s instructions with modifications. Only 1/3 of the recommended amount for all reagents is used in the NEBNext end prep and the adaptor ligation steps with a 1:10 dilution of the adaptor. A 30 ul PCR mixture (instead of the 50 ul reaction recommended) is divided into three PCR tubes for 10 cycles of amplification for the enrichment step.

The bulk tissue library construction is essentially the same as single crypt library construction except for 50 ng of purified bulk tissue DNA and an undiluted adaptor in the adaptor ligation reaction are used.

### Assessment and assembly of sequencing library

Detailed steps of assessing and assembling each colon crypt library and pooling of multiple libraries for each sequencing flow cell are summarized in Supplemental Fig. 1. Only one of the three PCR reactions is analyzed by BioAnalyzer (Model 2100, Agilent, Santa Clara, CA), and all three PCR reactions are quantitated by Qubit assay (Qubit 2.0 Fluorometer, Thermo Fisher Scientific). Each crypt-sequencing library is assembled by combining an equal quantity of library DNA from each of the three PCR tubes. The remaining DNA from each PCR tube is stored separately for variant verification in the future. The bulk tissue sequencing library is assembled by combining PCR products from all three tubes after BioAnalyzer analysis. All final sequencing libraries are purified with AmPure XP beads and evaluated by BioAnalyzer and Qubit analyses post-assembly.

A total of 81 crypt libraries and 16 bulk libraries are constructed and sequenced in six pools of up to 20 libraries per pool. Each pool is quality controlled by BioAnalyzer and qPCR analyses and shallow sequenced by MiSeq to verify adequate pooling.

### Sequencing and post-run quality assessment

Each library pool is diluted to a 0.7 nM final concentration and sequenced (150 bp paired-end) on an S4 flow cell using NovaSeq 6000 (Illumina, San Diego) S4 300 cycles reagent kit (v1.5) in the Keck Genomics Platform Core facility at USC. Post-sequencing, the read quality of sequencing reads is assessed by FastQC using BCL2FASTQ (v1.8.4).

Sequencing reads are aligned to GRCh38 by BWA (v0.7.8-r455), followed by GATK’s Base Recalibrator (v3.5.0) to detect quality score errors. Next, Picard Tools (v1.128) merges aligned BAMs and marks duplicate reads. GATK’s IndelRealigner minimizes mismatches across local alignments; Picard Tools GC Bias determines coverage bias; and Picard HS Metrics determines hybrid-selection metrics. Picard MultiMetrics and Samtools Stats (v1.2) collect multiple classes of metrics. VCFtools(v0.1.17) [9], Plinkv1.9 (v1.90b6.7) [10], SnpSniffer (v.7.0.0) (https://github.com/tgen/snpSniffer), and a variation of global ancestry principal component analysis [11] were used to analyze genome concordance between the bulk and crypt libraries for allele biasing.

## Results

All 97 libraries show a normal size distribution from 300 to 1000 bp with a peak of ~ 400 to 500 bp with no adaptor contamination and are of sufficient quantity when analyzed using BioAnalyzer (Supplemental Fig. 2). The yield of the 81 crypt libraries ranges from 69 ng to 685 ng with an average of 310 ng (Supplemental Fig. 3), within the 160 to 320 ng yield expected from the NEBNext Ultra kit for 6 ng DNA input with 10 PCR cycles of enrichment. NovaSeq 6000 (Illumina) generated > 120Gb of data per library. The libraries generated an average of 12 × 10^9^ clusters and 25 × 10^9^ reads at 91.6% >Q30 per run (Table 1). The overall reads generated from all 6 flow cells exceeded the Illumina recommended benchmark of a good quality sequencing run.

**Table 1:** Whole Genome Sequencing Performance on s$v1.5 Illumina NovaSeq 6000 2 × 150 bp

	PF Clusters	Paired-End Reads (B)	Yield (Gb)	%>Q30	Mean Quality
**Run 6**	12,913,455,779	26	3,489	90.8	35.4
**Run 5**	10,882,379,236	22	3,286	91.8	35.6
**Run 4**	12,811,554,090	26	3,869	91.0	35.4
**Run 3**	13,106,143,219	26	3,968	92.3	35.7
**Run 2**	12,401,208,646	25	3,745	91.8	35.6
**Run 1**	12,262,229,436	25	3,773	91.7	35.5
**Average**	12,396,161,734	25	3,688	91.6	35.5
Illumina Recommendations		16–20	2,400-3,000	> 85%	

**Fig. 2:**
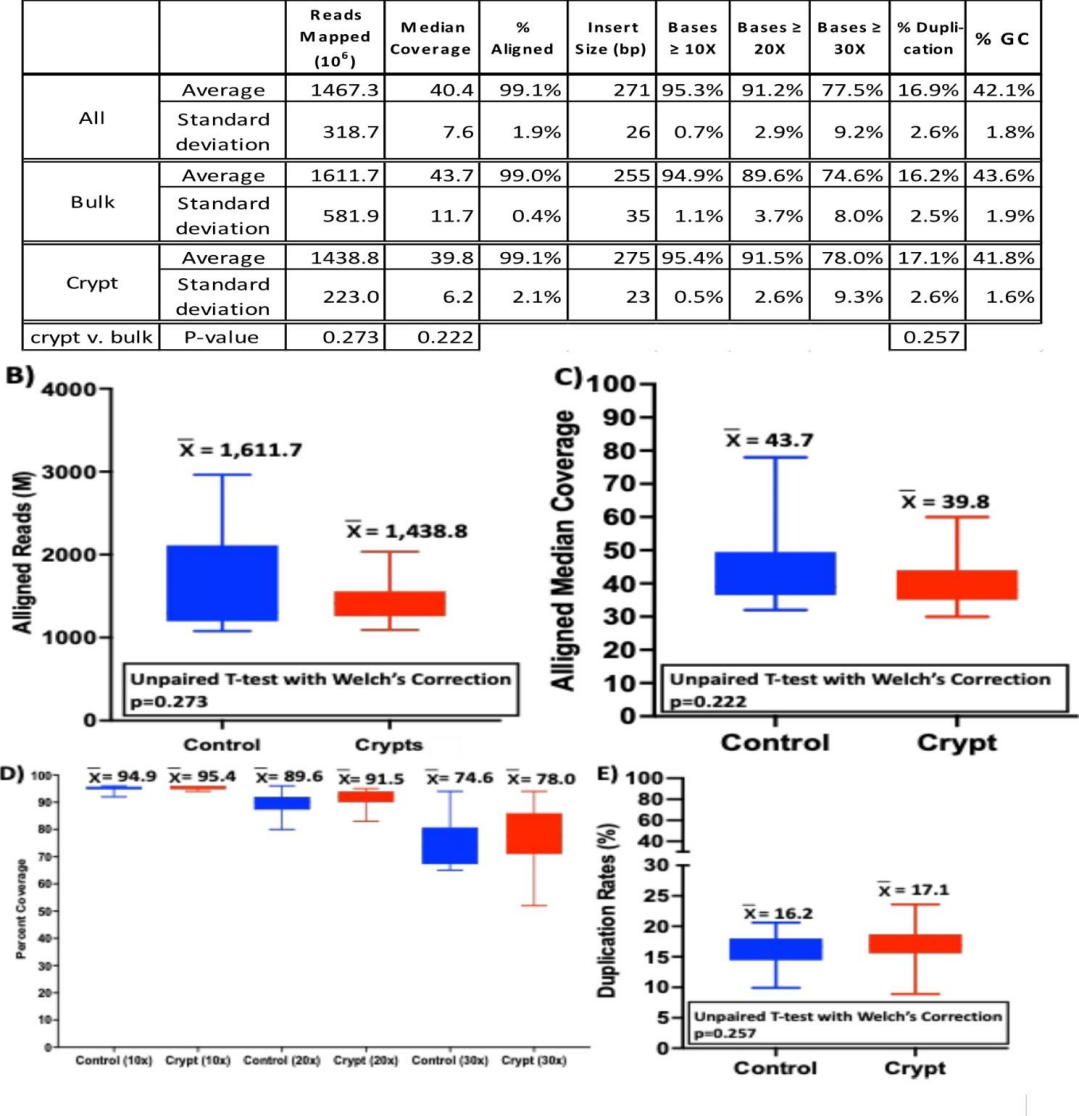
Post-alignment analysis of crypt and control (bulk) whole-genome libraries. (A) Summary of statistical analysis on all, bulk (control), and crypt libraries. Whisker plot presentation of (B) the number of aligned reads in millions (M); (C) median depth of coverage in multiples of genome; (D) percent of the genome covered with 10X, 20X, and 30X depth; and (E) duplication rates in percent of total aligned reads for bulk and crypt libraries. In B) through E), blue represents bulk libraries; red represents crypt libraries; the brackets indicate the range; and the average is shown above each bar

Next, the sequencing reads are aligned to the human genome and post-alignment statistics are collected (Supplemental Table 1). The performance of each set of crypt libraries is compared to a control library for the same patient. More than 1,100 × 10^6^ reads are mapped for each library, and there is no significant difference (p = 0.273) between the average number of reads per library generated from crypts and bulk controls (Fig. 2A and B). The percentage of aligned reads is 99% for both groups (Fig. 2A). The high mapping rates and the library insert size (Fig. 2A) indicate that these libraries are free from contamination and of good mapping quality.

Deep post-alignment genome coverage depth and broad coverage overall and at ≥ 10, ≥20, and ≥ 30 depth observed here indicate uniformity of the libraries and more than adequate usable data for reliable variant calling. The average median depth is 44X and 40X for bulk and crypt libraries, respectively, with no significant difference between the two groups (p = 0.222) (Fig. 2A and C). A similar average and standard deviation for the percentage of coverage across 10X, 20X, and 30X depth indicates sequencing uniformity within each group as well as between the two groups (Fig. 2A and D).

A high percentage of duplicate reads indicates low library diversity that can be caused by sample degradation, suboptimal sonication, inadequate adaptor ligation, and high PCR bias. The average duplication rate is 16.2% and 17.1% for bulk and crypt libraries, respectively, with no significant difference between the two groups (p = 0.257) (Fig. 2A and E). These duplication rates are within a workable range when using a low quantity of DNA as inputs [12]. The GC content, which is 41% for the human reference genome, reflects the balance of representation in a sequencing library. In our study, the average GC content is 43.6% and 41.8% of the bulk and crypt libraries, respectively (Fig. 2A).

Further SNP-based genome concordance analyses indicate no allele biasing in the crypt libraries. Plinkv1.9 analysis of > 5,000 SNPs shows an average concordance of 0.997 (Supplemental Table 2). Of 300 known heterozygous SNPs analyzed using SnpSniffer, bulk and crypt libraries from the same individual show an average of 0.998 concordance (Supplemental Table 3). Furthermore, principal component analysis also showed high genotype concordance estimated from 45,679 high-quality SNP distributed across autosomes between crypt and bulk libraries (Supplemental Table 4).

The comparisons described above clearly demonstrate that all 97 libraries are of good quality, and crypt libraries are comparable to bulk (control) libraries with no evidence of allele biasing.

## Discussion

We present a cost-effective method that consistently and reliably generates high-quality WGS libraries from as little as 1,000 to 2,000 cells, which is four- to 8-fold less than the purified DNA used in conventional sequencing library construction. Considering material loss during DNA extraction, our improvement is even more substantial. This improved method allows a comprehensive mutation analysis of a small number of cells and offers new possibilities for thorough genomic examination of material-limiting tissue sources. The key improvements are achieved by minimizing sample loss by constructing the library directly from the tissue without DNA extraction; optimizing the speed of completing each step; increasing reaction efficiency; and reducing PCR duplicates by dividing the enrichment PCR reaction into multiple reactions for 10 cycles of amplification.

We demonstrate that high-quality sequencing libraries could be made directly from < 2,000 cells without DNA extraction and that the quantity and size distribution of the library material generated are comparable to libraries constructed with ample purified DNA. All six sequencing runs outperformed Illumina’s recommended benchmark, and each library achieved greater than 30X genome coverage after removing duplicate reads post-alignment. Other studies successfully identified variants using microdissected colon crypts achieving a < 20X median depth coverage of the genome [8, 13]. All our crypt libraries, which exceed 31X median depth and cover > 90% of the genome at ≥ 15X depth, are more than sufficient for reliable variant calling. There is no significant difference in the depth of coverage and percentage of genome coverage between the 81 crypt libraries and the 16 control bulk libraries. Multiple analyses show no indication of allele biasing in the crypt libraries. We clearly show that the single crypt libraries are of comparable quality to libraries constructed from high quantities of purified DNA. Furthermore, our experimental design with three PCR reactions allows confirmation of whether any specific variant is an artifact from PCR or sequencing.

Our method can be applied to other tissue sources with minimal optimization and offers the possibility of interrogating small biopsy samples or limited tissue sources with depth and breadth of coverage equivalent to conventional WGS libraries. Our method can be used to profile variants in small numbers of cells, followed by targeted single-cell sequencing to obtain comprehensive cancer genome profiles and their evolution more cost-effectively and reliably than sequencing the whole genome of multiple single cells. An additional advantage of our method is that single-cell suspension is not required as the starting material; therefore, it can be applied to a broader selection of tissues. The potential broad applications of our method, without requiring any costly new equipment, would offer new possibilities in the comprehensive examination of genome heterogeneity in normal as well as diseased cells.

## Limitations

Our method is reliable, easily streamlined, and cost-effective without additional expensive technologies. It has possible applications across various tissue types with some needed optimizations for samples with a high lipid content or connective tissue. NEBNext Ultra II DNA Library Prep Kit can be used for our method, but the NEBNext Ultra II FS Library Prep Kit cannot be used because the DNA fragmentation step requires purified DNA.

## Electronic supplementary material

Below is the link to the electronic supplementary material.

Supplementary Material 1

## Data Availability

All data supporting the results of this article are included in this article and the supplementary information. The original alignment stats from PicardTools generated from the 97 sequencing libraries in this study are available from the corresponding author upon request.

## Abbreviations

WGS: Whole-genome sequencing
WGA: whole-genome amplification
