# Supplementary material for: Construction of high coverage whole-genome sequencing libraries from single colon crypts without DNA extraction or whole-genome amplification
Source: BMC Res Notes. 2023 Apr 27;16:66. doi: 10.1186/s13104-023-06333-y (PMC10142246; doi:10.1186/s13104-023-06333-y)
Supplement: Supplementary file 1 — Supplementary Material 1 [file 13104_2023_6333_MOESM1_ESM.pdf]

Supplemental Materials

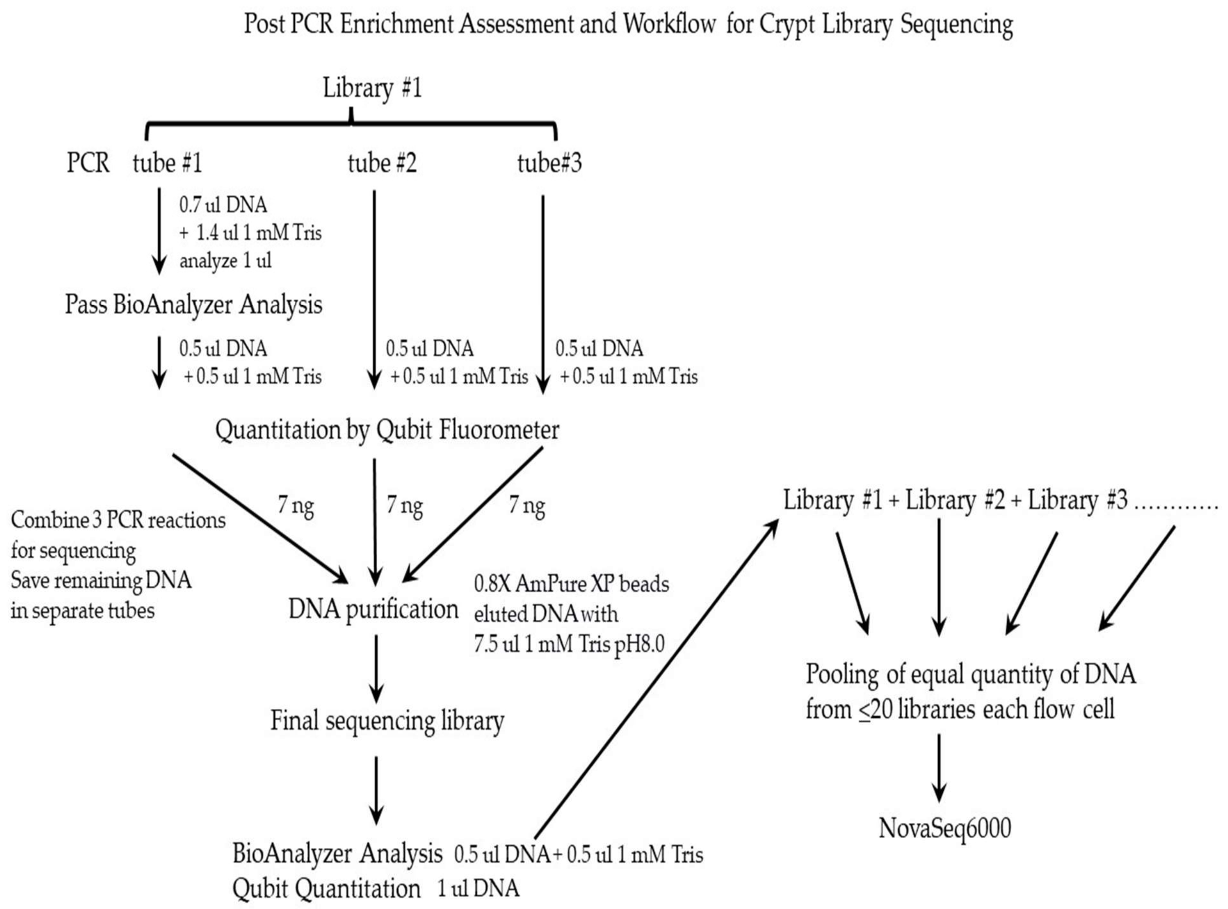

Figure S1 Workflow of post PCR quality assessment and library pooling for crypt libraries.

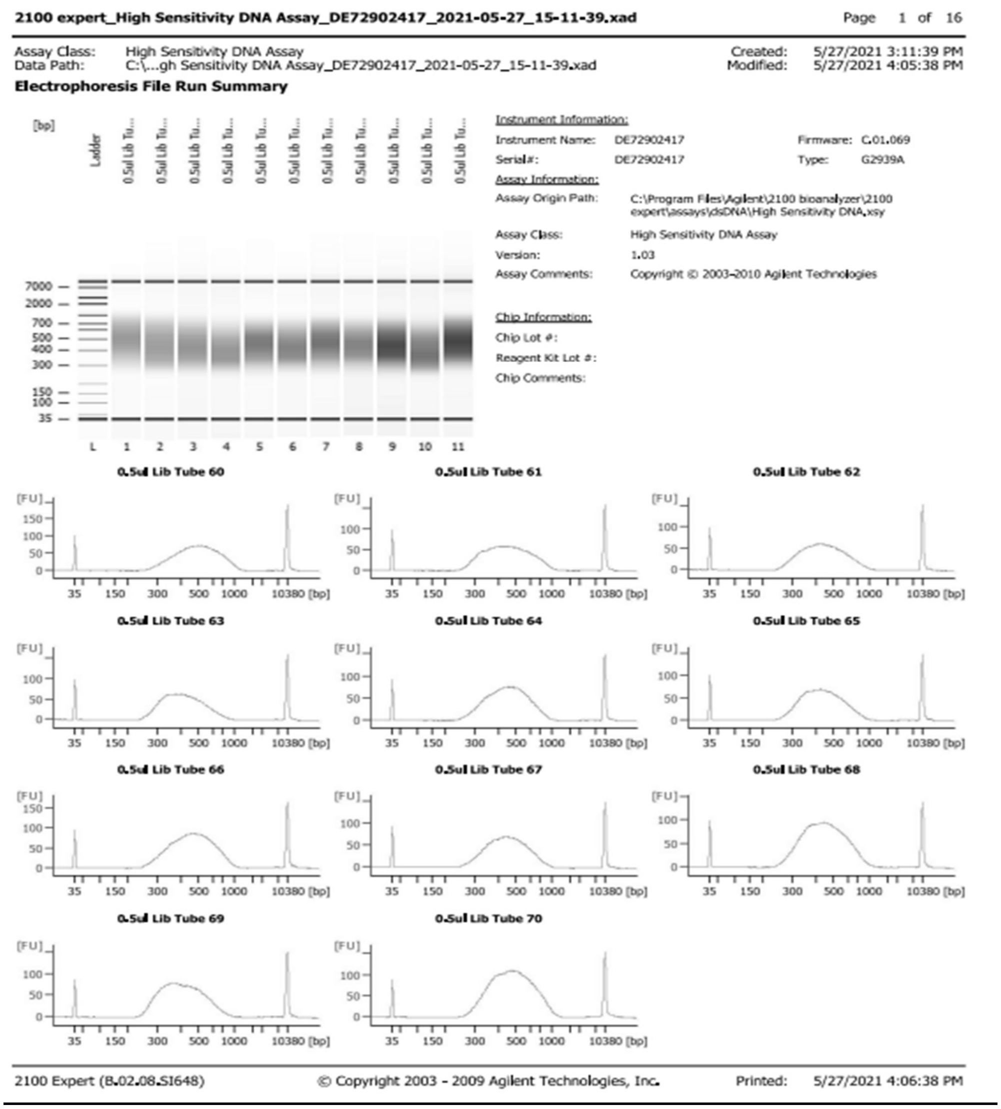

Figure S2 A representative assay output from BioAnalyzer.

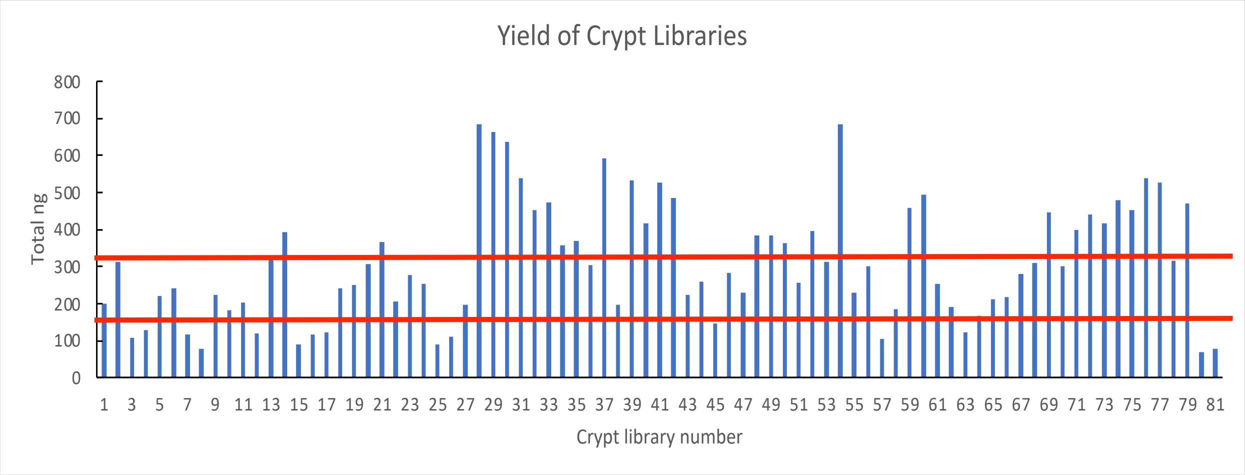

Figure S3 Total DNA quantity of 81 crypt libraries. Each bar presents the quantity of DNA yielded from each of the 81 crypt libraries. Each of the three PCR tubes from the same library contains 11.5 ul of DNA and quantity of the DNA in each PCR tube is calculated based on the quantitation of 0.5 ul of DNA from the specific PCR tube by Qubit assay. The total DNA quantity of the final crypt libraries is the sum of the amount of DNA in three PCR tubes of the same library. The two red horizontal lines indicate the expected range of yield (160 ng to 320 ng) from 6 ng of input DNA with 10 PCR cycles of enrichment using the NEBNext Ultra DNA ibrary Prep Kit according to the manufacturer.

Table S1 Whole Genome Sequencing Post-Allignment Output

Individual	Sample_IDs	Library type	Reads Mapped (x10^6)	% Aligned	Average Insert Size (bp)	Median Coverage	Bases ≥ 10X	Bases ≥ 20X	Bases ≥ 30X	% Duplica-tions	% GC
1	KUNUSCCLH_0001_LibTube01_T1	Crypt	1474.0	100%	304	42	95%	94%	90%	16%	40%
	KUNUSCCLH_0001_LibTube11_T2	Crypt	1411.2	100%	283	34	95%	92%	71%	19%	39%
	KUNUSCCLH_0001_LibTube19_T3	Crypt	1319.9	100%	309	39	95%	94%	86%	19%	40%
	KUNUSCCLH_0001_LibTube70_T4	Crypt	1440.7	100%	286	44	95%	94%	90%	15%	41%
	KUNUSCCLH_0001_LibTube77_T5	Crypt	1280.2	96%	285	40	95%	94%	84%	18%	43%
	KUNUSCCLH_0001_LibTube81_C1	Bulk	1277.1	99%	286	38	95%	90%	72%	17%	43%
2	KUNUSCCLH_0002_LibTube02_T1	Crypt	1519.1	100%	259	44	96%	93%	87%	11%	41%
	KUNUSCCLH_0002_LibTube05_T2	Crypt	1239.3	100%	268	37	95%	90%	73%	21%	42%
	KUNUSCCLH_0002_LibTube12_T3	Crypt	1706.0	100%	293	47	96%	94%	88%	18%	40%
	KUNUSCCLH_0002_LibTube26_T4	Crypt	1355.5	100%	241	34	94%	84%	63%	11%	42%
	KUNUSCCLH_0002_LibTube71_T5	Crypt	1288.2	100%	290	39	96%	92%	83%	19%	40%
	KUNUSCCLH_0002_LibTube82_C1	Bulk	1260.8	99%	285	39	96%	92%	82%	10%	41%
3	KUNUSCCLH_0003_LibTube06_T1	Crypt	1728.5	100%	245	48	95%	94%	90%	19%	42%
	KUNUSCCLH_0003_LibTube13_T2	Crypt	1431.4	100%	283	35	95%	93%	78%	15%	40%
	KUNUSCCLH_0003_LibTube20_T3	Crypt	1569.4	100%	318	47	95%	94%	92%	14%	40%
	KUNUSCCLH_0003_LibTube27_T4	Crypt	1769.6	100%	248	35	95%	92%	74%	18%	41%
	KUNUSCCLH_0003_LibTube28_T5	Crypt	1192.6	100%	297	35	95%	93%	76%	16%	40%
	KUNUSCCLH_0003_LibTube83_C1	Bulk	1149.6	99%	257	32	95%	92%	65%	17%	41%
4	KUNUSCCLH_0004_LibTube07_T1	Crypt	1500.3	100%	234	42	95%	93%	81%	16%	42%
	KUNUSCCLH_0004_LibTube14_T2	Crypt	1457.9	100%	282	45	95%	94%	91%	18%	41%
	KUNUSCCLH_0004_LibTube21_T3	Crypt	1156.0	100%	286	35	95%	93%	76%	15%	40%
	KUNUSCCLH_0004_LibTube29_T4	Crypt	1178.6	100%	265	36	95%	92%	72%	15%	42%
	KUNUSCCLH_0004_LibTube72_T5	Crypt	1254.7	100%	278	38	95%	94%	83%	16%	41%
	KUNUSCCLH_0004_LibTube84_C1	Bulk	1080.0	99%	278	33	95%	92%	67%	17%	41%
5	KUNUSCCLH_0005_LibTube08_T1	Crypt	1789.2	100%	252	45	96%	93%	86%	19%	41%
	KUNUSCCLH_0005_LibTube15_T2	Crypt	1252.8	100%	302	39	96%	92%	82%	16%	40%
	KUNUSCCLH_0005_LibTube22_T3	Crypt	1347.5	100%	258	40	96%	91%	78%	18%	42%
	KUNUSCCLH_0005_LibTube30_T4	Crypt	1930.1	100%	247	51	96%	94%	90%	14%	42%
	KUNUSCCLH_0005_LibTube42_T5	Crypt	2008.1	100%	278	60	96%	95%	92%	19%	42%
	KUNUSCCLH_0005_LibTube85_C1	Bulk	1349.9	99%	302	41	95%	90%	75%	16%	44%
6	KUNUSCCLH_0006_LibTube16_T1	Crypt	1478.3	100%	282	33	95%	89%	66%	24%	40%
	KUNUSCCLH_0006_LibTube23_T2	Crypt	1732.5	98%	322	53	96%	95%	90%	19%	41%
	KUNUSCCLH_0006_LibTube31_T3	Crypt	1655.3	100%	247	49	96%	94%	89%	12%	42%
	KUNUSCCLH_0006_LibTube43_T4	Crypt	1525.2	100%	277	46	96%	93%	86%	16%	42%
	KUNUSCCLH_0006_LibTube78_T5	Crypt	1401.5	95%	280	43	96%	93%	86%	18%	43%
	KUNUSCCLH_0006_LibTube86_C1	Bulk	1319.4	99%	259	39	96%	91%	76%	21%	43%
7	KUNUSCCLH_0007_LibTube09_T1	Crypt	1687.1	100%	254	44	96%	91%	80%	21%	42%
	KUNUSCCLH_0007_LibTube17_T2	Crypt	1318.7	100%	289	35	95%	90%	73%	16%	40%
	KUNUSCCLH_0007_LibTube24_T3	Crypt	1259.8	100%	273	39	96%	91%	79%	15%	42%
	KUNUSCCLH_0007_LibTube32_T4	Crypt	1499.4	100%	243	43	96%	92%	83%	19%	42%
	KUNUSCCLH_0007_LibTube55_T5	Crypt	1093.3	100%	269	34	95%	88%	65%	15%	42%
	KUNUSCCLH_0007_LibTube87_C1	Bulk	1737.2	99%	254	51	96%	94%	87%	20%	43%
8	KUNUSCCLH_0008_LibTube10_T1	Crypt	1473.4	100%	235	38	95%	88%	71%	18%	43%
	KUNUSCCLH_0008_LibTube18_T2	Crypt	1398.5	100%	273	33	95%	90%	69%	18%	40%
	KUNUSCCLH_0008_LibTube25_T3	Crypt	1464.6	100%	292	46	96%	93%	86%	14%	42%
	KUNUSCCLH_0008_LibTube33_T4	Crypt	1655.6	100%	261	48	96%	94%	88%	17%	42%
	KUNUSCCLH_0008_LibTube62_T5	Crypt	1455.1	100%	247	39	95%	90%	75%	14%	43%
	KUNUSCCLH_0008_LibTube88_C1	Bulk	1279.3	99%	263	39	95%	90%	74%	14%	43%
9	KUNUSCCLH_0009_LibTube34_T1	Crypt	1118.7	100%	306	34	95%	90%	71%	17%	41%
	KUNUSCCLH_0009_LibTube46_T2	Crypt	1333.6	99%	280	31	95%	89%	59%	14%	47%
	KUNUSCCLH_0009_LibTube56_T3	Crypt	1132.0	100%	301	32	95%	89%	63%	15%	40%
	KUNUSCCLH_0009_LibTube63_T4	Crypt	1814.7	100%	231	45	95%	89%	77%	20%	44%
	KUNUSCCLH_0009_LibTube73_T5	Crypt	1364.7	100%	277	37	96%	91%	80%	15%	41%
	KUNUSCCLH_0009_LibTube89_C1	Bulk	1108.8	99%	285	36	95%	90%	73%	17%	42%
10	KUNUSCCLH_0010_LibTube35_T1	Crypt	2035.0	100%	312	58	96%	95%	92%	20%	41%
	KUNUSCCLH_0010_LibTube47_T2	Crypt	1912.3	99%	272	49	96%	94%	89%	19%	41%
	KUNUSCCLH_0010_LibTube64_T3	Crypt	1334.4	99%	259	35	95%	85%	64%	16%	43%
	KUNUSCCLH_0010_LibTube79_T4	Crypt	1150.7	93%	293	34	95%	90%	71%	18%	42%
	KUNUSCCLH_0010_LibTube80_T5	Crypt	1340.6	94%	285	40	96%	92%	83%	17%	42%
	KUNUSCCLH_0010_LibTube90_C1	Bulk	1194.1	99%	259	36	95%	87%	68%	15%	43%
11	KUNUSCCLH_0011_LibTube36_T1	Crypt	1242.9	100%	306	38	96%	91%	80%	16%	41%
	KUNUSCCLH_0011_LibTube48_T2	Crypt	1127.1	99%	302	33	95%	89%	66%	16%	41%
	KUNUSCCLH_0011_LibTube52_T3	Crypt	1480.1	100%	251	39	96%	91%	77%	17%	42%
	KUNUSCCLH_0011_LibTube57_T4	Crypt	1182.9	100%	305	37	96%	91%	78%	19%	41%
	KUNUSCCLH_0011_LibTube65_T5	Crypt	1455.5	100%	248	37	95%	88%	69%	20%	43%
	KUNUSCCLH_0011_LibTube91_C1	Bulk	2519.7	99%	268	78	96%	96%	94%	18%	44%
12	KUNUSCCLH_0012_LibTube03_T1	Crypt	1214.1	100%	276	34	95%	90%	71%	17%	41%
	KUNUSCCLH_0012_LibTube37_T3	Crypt	1182.8	100%	291	37	96%	91%	76%	20%	42%
	KUNUSCCLH_0012_LibTube49_T4	Crypt	1195.0	100%	286	35	96%	91%	75%	17%	41%
	KUNUSCCLH_0012_LibTube53_T5	Crypt	1294.0	100%	245	35	95%	86%	65%	16%	43%
	KUNUSCCLH_0012_LibTube98_T6	Crypt	1618.1	93%	240	37	96%	90%	74%	20%	44%
	KUNUSCCLH_0012_LibTube99_T7	Crypt	1639.3	93%	257	34	95%	89%	69%	20%	44%
	KUNUSCCLH_0012_LibTube92_C1	Bulk	1202.7	100%	284	38	95%	88%	69%	13%	44%
13	KUNUSCCLH_0013_LibTube38_T1	Crypt	1186.5	100%	306	38	95%	93%	82%	17%	41%
	KUNUSCCLH_0013_LibTube50_T2	Crypt	1242.7	100%	281	36	95%	94%	80%	16%	41%
	KUNUSCCLH_0013_LibTube58_T3	Crypt	1865.0	99%	283	30	95%	91%	52%	21%	51%
	KUNUSCCLH_0013_LibTube66_T4	Crypt	1291.3	100%	263	36	95%	90%	71%	16%	42%
	KUNUSCCLH_0013_LibTube74_T5	Crypt	1831.7	100%	272	54	95%	95%	94%	16%	41%
	KUNUSCCLH_0013_LibTube93_C1	Bulk	1617.8	98%	210	38	94%	85%	66%	17%	45%
14	KUNUSCCLH_0014_LibTube39_T1	Crypt	1335.3	92%	299	34	95%	93%	75%	9%	43%
	KUNUSCCLH_0014_LibTube51_T2	Crypt	1526.1	96%	294	44	95%	94%	91%	16%	42%
	KUNUSCCLH_0014_LibTube54_T3	Crypt	1403.6	95%	260	34	95%	92%	71%	21%	43%
	KUNUSCCLH_0014_LibTube59_T4	Crypt	1521.3	93%	314	35	95%	94%	78%	23%	42%
	KUNUSCCLH_0014_LibTube67_T5	Crypt	1383.9	94%	256	32	95%	89%	62%	13%	43%
	KUNUSCCLH_0014_LibTube94_C1	Bulk	2962.1	99%	191	60	93%	86%	77%	14%	47%
15	KUNUSCCLH_0015_LibTube40_T1	Crypt	1471.9	100%	282	45	96%	93%	84%	15%	42%
	KUNUSCCLH_0015_LibTube44_T2	Crypt	1606.3	99%	272	42	96%	93%	86%	21%	41%
	KUNUSCCLH_0015_LibTube60_T3	Crypt	1617.9	100%	291	47	96%	94%	88%	16%	41%
	KUNUSCCLH_0015_LibTube68_T4	Crypt	1319.8	100%	257	36	95%	86%	67%	17%	43%
	KUNUSCCLH_0015_LibTube75_T5	Crypt	1417.8	100%	242	41	95%	91%	78%	17%	43%
	KUNUSCCLH_0015_LibTube95_C1	Bulk	2490.2	99%	192	56	95%	91%	82%	18%	46%
16	KUNUSCCLH_0016_LibTube41_T1	Crypt	1260.6	99%	301	38	96%	91%	79%	17%	41%
	KUNUSCCLH_0016_LibTube45_T2	Crypt	1178.2	99%	272	33	95%	89%	65%	17%	41%
	KUNUSCCLH_0016_LibTube61_T3	Crypt	1215.4	100%	252	37	95%	90%	72%	16%	42%
	KUNUSCCLH_0016_LibTube69_T4	Crypt	1456.5	100%	223	37	94%	83%	65%	19%	44%
	KUNUSCCLH_0016_LibTube76_T5	Crypt	1546.0	100%	277	46	96%	94%	88%	22%	41%
	KUNUSCCLH_0016_LibTube96_C1	Bulk	2237.8	99%	199	45	92%	80%	67%	18%	47%

Table S2 Genotyping relatness between crypt vs. bulk using Plinkv1.9.

Bulk_ID	Crypt_ID	DST	PPC	Z2	PI_HAT
KUNUSCCLH_0001_01_LibTube81_R00250S8A2M0000P0000_C1_KHWGSH_A00006	KUNUSCCLH_0001_01_LibTube01_R00250S8A2M0000P0000_T1_KHWGSH_A00001	0.999821	1	0.999	0.9995
KUNUSCCLH_0001_01_LibTube81_R00250S8A2M0000P0000_C1_KHWGSH_A00006	KUNUSCCLH_0001_01_LibTube11_R00250S8A2M0000P0000_T2_KHWGSH_A00002	0.999464	1	0.9969	0.9985
KUNUSCCLH_0001_01_LibTube81_R00250S8A2M0000P0000_C1_KHWGSH_A00006	KUNUSCCLH_0001_01_LibTube19_R00250S8A2M0000P0000_T3_KHWGSH_A00003	0.999285	1	0.9959	0.9979
KUNUSCCLH_0001_01_LibTube81_R00250S8A2M0000P0000_C1_KHWGSH_A00006	KUNUSCCLH_0001_01_LibTube70_R00250S8A2M0000P0000_T4_KHWGSH_A00004	0.999643	1	0.9979	0.999
KUNUSCCLH_0001_01_LibTube81_R00250S8A2M0000P0000_C1_KHWGSH_A00006	KUNUSCCLH_0001_01_LibTube77_R00250S8A2M0000P0000_T5_KHWGSH_A00005	0.999464	1	0.9949	0.9949
KUNUSCCLH_0002_01_LibTube82_R00250S8A2M0000P0000_C1_KHWGSH_A00012	KUNUSCCLH_0002_01_LibTube12_R00250S8A2M0000P0000_T3_KHWGSH_A00009	0.999821	1	0.999	0.9995
KUNUSCCLH_0002_01_LibTube82_R00250S8A2M0000P0000_C1_KHWGSH_A00012	KUNUSCCLH_0002_01_LibTube26_R00250S8A2M0000P0000_T4_KHWGSH_A00010	0.999107	1	0.9949	0.9949
KUNUSCCLH_0002_01_LibTube82_R00250S8A2M0000P0000_C1_KHWGSH_A00012	KUNUSCCLH_0002_01_LibTube2_R00250S8A2M0000P0000_T1_KHWGSH_A00007	0.998749	1	0.9949	0.9949
KUNUSCCLH_0002_01_LibTube82_R00250S8A2M0000P0000_C1_KHWGSH_A00012	KUNUSCCLH_0002_01_LibTube5_R00250S8A2M0000P0000_T2_KHWGSH_A00008	0.998928	1	0.9949	0.9949
KUNUSCCLH_0002_01_LibTube82_R00250S8A2M0000P0000_C1_KHWGSH_A00012	KUNUSCCLH_0002_01_LibTube71_R00250S8A2M0000P0000_T5_KHWGSH_A00011	0.999107	1	0.9949	0.9949
KUNUSCCLH_0003_01_LibTube83_R00250S8A2M0000P0000_C1_KHWGSH_A00018	KUNUSCCLH_0003_01_LibTube13_R00250S8A2M0000P0000_T2_KHWGSH_A00014	0.999464	1	0.9969	0.9985
KUNUSCCLH_0003_01_LibTube83_R00250S8A2M0000P0000_C1_KHWGSH_A00018	KUNUSCCLH_0003_01_LibTube20_R00250S8A2M0000P0000_T3_KHWGSH_A00015	0.999285	1	0.9959	0.9979
KUNUSCCLH_0003_01_LibTube83_R00250S8A2M0000P0000_C1_KHWGSH_A00018	KUNUSCCLH_0003_01_LibTube27_R00250S8A2M0000P0000_T4_KHWGSH_A00016	0.997855	1	0.9877	0.9938
KUNUSCCLH_0003_01_LibTube83_R00250S8A2M0000P0000_C1_KHWGSH_A00018	KUNUSCCLH_0003_01_LibTube28_R00250S8A2M0000P0000_T5_KHWGSH_A00017	0.999642	1	0.9979	0.999
KUNUSCCLH_0003_01_LibTube83_R00250S8A2M0000P0000_C1_KHWGSH_A00018	KUNUSCCLH_0003_01_LibTube6_R00250S8A2M0000P0000_T1_KHWGSH_A00013	0.999464	1	0.9969	0.9985
KUNUSCCLH_0004_01_LibTube84_R00250S8A2M0000P0000_C1_KHWGSH_A00024	KUNUSCCLH_0004_01_LibTube07_R00250S8A2M0000P0000_T1_KHWGSH_A00019	0.999464	1	0.9969	0.9985
KUNUSCCLH_0004_01_LibTube84_R00250S8A2M0000P0000_C1_KHWGSH_A00024	KUNUSCCLH_0004_01_LibTube14_R00250S8A2M0000P0000_T2_KHWGSH_A00020	0.999285	1	0.9959	0.9979
KUNUSCCLH_0004_01_LibTube84_R00250S8A2M0000P0000_C1_KHWGSH_A00024	KUNUSCCLH_0004_01_LibTube21_R00250S8A2M0000P0000_T3_KHWGSH_A00021	0.999285	1	0.9959	0.9979
KUNUSCCLH_0004_01_LibTube84_R00250S8A2M0000P0000_C1_KHWGSH_A00024	KUNUSCCLH_0004_01_LibTube29_R00250S8A2M0000P0000_T4_KHWGSH_A00022	0.998927	1	0.9938	0.9969
KUNUSCCLH_0004_01_LibTube84_R00250S8A2M0000P0000_C1_KHWGSH_A00024	KUNUSCCLH_0004_01_LibTube72_R00250S8A2M0000P0000_T5_KHWGSH_A00023	0.999107	1	0.9949	0.9974
KUNUSCCLH_0005_01_LibTube85_R00250S8A2M0000P0000_C1_KHWGSH_A00030	KUNUSCCLH_0005_01_LibTube08_R00250S8A2M0000P0000_T1_KHWGSH_A00025	0.99857	1	0.9918	0.9959
KUNUSCCLH_0005_01_LibTube85_R00250S8A2M0000P0000_C1_KHWGSH_A00030	KUNUSCCLH_0005_01_LibTube15_R00250S8A2M0000P0000_T2_KHWGSH_A00026	0.999821	1	0.999	0.9995
KUNUSCCLH_0005_01_LibTube85_R00250S8A2M0000P0000_C1_KHWGSH_A00030	KUNUSCCLH_0005_01_LibTube22_R00250S8A2M0000P0000_T3_KHWGSH_A00027	0.999642	1	0.9979	0.999
KUNUSCCLH_0005_01_LibTube85_R00250S8A2M0000P0000_C1_KHWGSH_A00030	KUNUSCCLH_0005_01_LibTube30_R00250S8A2M0000P0000_T4_KHWGSH_A00028	0.999464	1	0.9969	0.9985
KUNUSCCLH_0005_01_LibTube85_R00250S8A2M0000P0000_C1_KHWGSH_A00030	KUNUSCCLH_0005_01_LibTube42_R00250S8A2M0000P0000_T5_KHWGSH_A00029	0.999285	1	0.9949	0.9949
KUNUSCCLH_0006_01_LibTube86_R00250S8A2M0000P0000_C1_KHWGSH_A00036	KUNUSCCLH_0006_01_LibTube16_R00250S8A2M0000P0000_T1_KHWGSH_A00031	0.999464	1	0.9969	0.9985
KUNUSCCLH_0006_01_LibTube86_R00250S8A2M0000P0000_C1_KHWGSH_A00036	KUNUSCCLH_0006_01_LibTube23_R00250S8A2M0000P0000_T2_KHWGSH_A00032	0.998928	1	0.9949	0.9949
KUNUSCCLH_0006_01_LibTube86_R00250S8A2M0000P0000_C1_KHWGSH_A00036	KUNUSCCLH_0006_01_LibTube31_R00250S8A2M0000P0000_T3_KHWGSH_A00033	0.999107	1	0.9949	0.9949
KUNUSCCLH_0006_01_LibTube86_R00250S8A2M0000P0000_C1_KHWGSH_A00036	KUNUSCCLH_0006_01_LibTube43_R00250S8A2M0000P0000_T4_KHWGSH_A00034	0.999107	1	0.9949	0.9974
KUNUSCCLH_0006_01_LibTube86_R00250S8A2M0000P0000_C1_KHWGSH_A00036	KUNUSCCLH_0006_01_LibTube78_R00250S8A2M0000P0000_T5_KHWGSH_A00035	0.999464	1	0.9969	0.9985
KUNUSCCLH_0007_01_LibTube87_R00250S8A2M0000P0000_C1_KHWGSH_A00042	KUNUSCCLH_0007_01_LibTube9_R00250S8A2M0000P0000_T1_KHWGSH_A00037	0.999464	1	0.9969	0.9985
KUNUSCCLH_0007_01_LibTube87_R00250S8A2M0000P0000_C1_KHWGSH_A00042	KUNUSCCLH_0007_01_LibTube17_R00250S8A2M0000P0000_T2_KHWGSH_A00038	0.999285	1	0.9959	0.9979
KUNUSCCLH_0007_01_LibTube87_R00250S8A2M0000P0000_C1_KHWGSH_A00042	KUNUSCCLH_0007_01_LibTube24_R00250S8A2M0000P0000_T3_KHWGSH_A00039	0.999107	1	0.9949	0.9949
KUNUSCCLH_0007_01_LibTube87_R00250S8A2M0000P0000_C1_KHWGSH_A00042	KUNUSCCLH_0007_01_LibTube32_R00250S8A2M0000P0000_T4_KHWGSH_A00040	0.999464	1	0.9969	0.9985
KUNUSCCLH_0007_01_LibTube87_R00250S8A2M0000P0000_C1_KHWGSH_A00042	KUNUSCCLH_0007_01_LibTube55_R00250S8A2M0000P0000_T5_KHWGSH_A00041	0.99875	1	0.9928	0.9964
KUNUSCCLH_0008_01_LibTube88_R00250S8A2M0000P0000_C1_KHWGSH_A00048	KUNUSCCLH_0008_01_LibTube10_R00250S8A2M0000P0000_T1_KHWGSH_A00043	0.999106	1	0.9949	0.9974
KUNUSCCLH_0008_01_LibTube88_R00250S8A2M0000P0000_C1_KHWGSH_A00048	KUNUSCCLH_0008_01_LibTube18_R00250S8A2M0000P0000_T2_KHWGSH_A00044	0.998569	1	0.9948	0.9949
KUNUSCCLH_0008_01_LibTube88_R00250S8A2M0000P0000_C1_KHWGSH_A00048	KUNUSCCLH_0008_01_LibTube25_R00250S8A2M0000P0000_T3_KHWGSH_A00045	0.998392	1	0.9899	0.9899
KUNUSCCLH_0008_01_LibTube88_R00250S8A2M0000P0000_C1_KHWGSH_A00048	KUNUSCCLH_0008_01_LibTube33_R00250S8A2M0000P0000_T4_KHWGSH_A00046	0.998749	1	0.9949	0.9949
KUNUSCCLH_0008_01_LibTube88_R00250S8A2M0000P0000_C1_KHWGSH_A00048	KUNUSCCLH_0008_01_LibTube62_R00250S8A2M0000P0000_T5_KHWGSH_A00047	0.998927	1	0.9938	0.9969
KUNUSCCLH_0009_01_LibTube89_R00250S8A2M0000P0000_C1_KHWGSH_A00054	KUNUSCCLH_0009_01_LibTube34_R00250S8A2M0000P0000_T1_KHWGSH_A00049	0.999464	1	0.9969	0.9985
KUNUSCCLH_0009_01_LibTube89_R00250S8A2M0000P0000_C1_KHWGSH_A00054	KUNUSCCLH_0009_01_LibTube46_R00250S8A2M0000P0000_T2_KHWGSH_A00050	0.999643	1	0.9979	0.999
KUNUSCCLH_0009_01_LibTube89_R00250S8A2M0000P0000_C1_KHWGSH_A00054	KUNUSCCLH_0009_01_LibTube56_R00250S8A2M0000P0000_T3_KHWGSH_A00051	0.999464	1	0.9969	0.9985
KUNUSCCLH_0009_01_LibTube89_R00250S8A2M0000P0000_C1_KHWGSH_A00054	KUNUSCCLH_0009_01_LibTube63_R00250S8A2M0000P0000_T4_KHWGSH_A00052	0.998749	1	0.9899	0.9899
KUNUSCCLH_0009_01_LibTube89_R00250S8A2M0000P0000_C1_KHWGSH_A00054	KUNUSCCLH_0009_01_LibTube73_R00250S8A2M0000P0000_T5_KHWGSH_A00053	0.998212	1	0.9898	0.9898
KUNUSCCLH_0010_01_LibTube90_R00250S8A2M0000P0000_C1_KHWGSH_A00060	KUNUSCCLH_0010_01_LibTube35_R00250S8A2M0000P0000_T1_KHWGSH_A00055	1	1	1	1
KUNUSCCLH_0010_01_LibTube90_R00250S8A2M0000P0000_C1_KHWGSH_A00060	KUNUSCCLH_0010_01_LibTube47_R00250S8A2M0000P0000_T2_KHWGSH_A00056	0.998928	1	0.9949	0.9949
KUNUSCCLH_0010_01_LibTube90_R00250S8A2M0000P0000_C1_KHWGSH_A00060	KUNUSCCLH_0010_01_LibTube64_R00250S8A2M0000P0000_T3_KHWGSH_A00057	0.999464	1	0.9969	0.9985
KUNUSCCLH_0010_01_LibTube90_R00250S8A2M0000P0000_C1_KHWGSH_A00060	KUNUSCCLH_0010_01_LibTube79_R00250S8A2M0000P0000_T4_KHWGSH_A00058	0.999464	1	0.9969	0.9985
KUNUSCCLH_0010_01_LibTube90_R00250S8A2M0000P0000_C1_KHWGSH_A00060	KUNUSCCLH_0010_01_LibTube80_R00250S8A2M0000P0000_T5_KHWGSH_A00059	0.999464	1	0.9969	0.9985
KUNUSCCLH_0011_01_LibTube91_R00250S8A2M0000P0000_C1_KHWGSH_A00066	KUNUSCCLH_0011_01_LibTube36_R00250S8A2M0000P0000_T1_KHWGSH_A00061	0.999464	1	0.9969	0.9985
KUNUSCCLH_0011_01_LibTube91_R00250S8A2M0000P0000_C1_KHWGSH_A00066	KUNUSCCLH_0011_01_LibTube48_R00250S8A2M0000P0000_T2_KHWGSH_A00062	0.999464	1	0.9969	0.9985
KUNUSCCLH_0011_01_LibTube91_R00250S8A2M0000P0000_C1_KHWGSH_A00066	KUNUSCCLH_0011_01_LibTube52_R00250S8A2M0000P0000_T3_KHWGSH_A00063	0.999821	1	0.999	0.9995
KUNUSCCLH_0011_01_LibTube91_R00250S8A2M0000P0000_C1_KHWGSH_A00066	KUNUSCCLH_0011_01_LibTube57_R00250S8A2M0000P0000_T4_KHWGSH_A00064	0.999285	1	0.9959	0.9979
KUNUSCCLH_0011_01_LibTube91_R00250S8A2M0000P0000_C1_KHWGSH_A00066	KUNUSCCLH_0011_01_LibTube65_R00250S8A2M0000P0000_T5_KHWGSH_A00065	0.999821	1	0.999	0.9995
KUNUSCCLH_0012_01_LibTube92_R00250S8A2M0000P0000_C1_KHWGSH_A00072	KUNUSCCLH_0012_01_LibTube37_R00250S8A2M0000P0000_T3_KHWGSH_A00069	0.999821	1	0.999	0.9995
KUNUSCCLH_0012_01_LibTube92_R00250S8A2M0000P0000_C1_KHWGSH_A00072	KUNUSCCLH_0012_01_LibTube3_R00250S8A2M0000P0000_T1_KHWGSH_A00067	0.999107	1	0.9949	0.9949
KUNUSCCLH_0012_01_LibTube92_R00250S8A2M0000P0000_C1_KHWGSH_A00072	KUNUSCCLH_0012_01_LibTube49_R00250S8A2M0000P0000_T4_KHWGSH_A00070	0.998392	1	0.9938	0.9943
KUNUSCCLH_0012_01_LibTube92_R00250S8A2M0000P0000_C1_KHWGSH_A00072	KUNUSCCLH_0012_01_LibTube53_R00250S8A2M0000P0000_T5_KHWGSH_A00071	0.998571	1	0.9899	0.9899
KUNUSCCLH_0012_01_LibTube92_R00250S8A2M0000P0000_C1_KHWGSH_A00072	KUNUSCCLH_0012_01_LibTube98_R00250S8A2M0000P0000_T6_KHWGSH_A00097	0.998214	1	0.9897	0.9949
KUNUSCCLH_0012_01_LibTube92_R00250S8A2M0000P0000_C1_KHWGSH_A00072	KUNUSCCLH_0012_01_LibTube99_R00250S8A2M0000P0000_T7_KHWGSH_A00098	0.998392	1	0.9938	0.9943
KUNUSCCLH_0013_01_LibTube93_R00250S8A2M0000P0000_C1_KHWGSH_A00078	KUNUSCCLH_0013_01_LibTube38_R00250S8A2M0000P0000_T1_KHWGSH_A00073	0.999464	1	0.9969	0.9985
KUNUSCCLH_0013_01_LibTube93_R00250S8A2M0000P0000_C1_KHWGSH_A00078	KUNUSCCLH_0013_01_LibTube50_R00250S8A2M0000P0000_T2_KHWGSH_A00074	0.999464	1	0.9969	0.9985
KUNUSCCLH_0013_01_LibTube93_R00250S8A2M0000P0000_C1_KHWGSH_A00078	KUNUSCCLH_0013_01_LibTube58_R00250S8A2M0000P0000_T3_KHWGSH_A00075	0.998749	1	0.9928	0.9964
KUNUSCCLH_0013_01_LibTube93_R00250S8A2M0000P0000_C1_KHWGSH_A00078	KUNUSCCLH_0013_01_LibTube66_R00250S8A2M0000P0000_T4_KHWGSH_A00076	0.999107	1	0.9949	0.9974
KUNUSCCLH_0013_01_LibTube93_R00250S8A2M0000P0000_C1_KHWGSH_A00078	KUNUSCCLH_0013_01_LibTube74_R00250S8A2M0000P0000_T5_KHWGSH_A00077	0.998928	1	0.9938	0.9969
KUNUSCCLH_0014_01_LibTube94_R00250S8A2M0000P0000_C1_KHWGSH_A00084	KUNUSCCLH_0014_01_LibTube39_R00250S8A2M0000P0000_T1_KHWGSH_A00079	0.998928	1	0.9938	0.9969
KUNUSCCLH_0014_01_LibTube94_R00250S8A2M0000P0000_C1_KHWGSH_A00084	KUNUSCCLH_0014_01_LibTube51_R00250S8A2M0000P0000_T2_KHWGSH_A00080	0.999464	1	0.9969	0.9985
KUNUSCCLH_0014_01_LibTube94_R00250S8A2M0000P0000_C1_KHWGSH_A00084	KUNUSCCLH_0014_01_LibTube54_R00250S8A2M0000P0000_T3_KHWGSH_A00081	0.999464	1	0.9969	0.9985
KUNUSCCLH_0014_01_LibTube94_R00250S8A2M0000P0000_C1_KHWGSH_A00084	KUNUSCCLH_0014_01_LibTube59_R00250S8A2M0000P0000_T4_KHWGSH_A00082	0.999464	1	0.9969	0.9985
KUNUSCCLH_0014_01_LibTube94_R00250S8A2M0000P0000_C1_KHWGSH_A00084	KUNUSCCLH_0014_01_LibTube67_R00250S8A2M0000P0000_T5_KHWGSH_A00083	0.999464	1	0.9969	0.9985
KUNUSCCLH_0015_01_LibTube95_R00250S8A2M0000P0000_C1_KHWGSH_A00090	KUNUSCCLH_0015_01_LibTube40_R00250S8A2M0000P0000_T1_KHWGSH_A00085	0.998928	1	0.9938	0.9969
KUNUSCCLH_0015_01_LibTube95_R00250S8A2M0000P0000_C1_KHWGSH_A00090	KUNUSCCLH_0015_01_LibTube44_R00250S8A2M0000P0000_T2_KHWGSH_A00086	0.998749	1	0.9928	0.9964
KUNUSCCLH_0015_01_LibTube95_R00250S8A2M0000P0000_C1_KHWGSH_A00090	KUNUSCCLH_0015_01_LibTube60_R00250S8A2M0000P0000_T3_KHWGSH_A00087	0.999107	1	0.9949	0.9974
KUNUSCCLH_0015_01_LibTube95_R00250S8A2M0000P0000_C1_KHWGSH_A00090	KUNUSCCLH_0015_01_LibTube68_R00250S8A2M0000P0000_T4_KHWGSH_A00088	0.998928	1	0.9949	0.9949
KUNUSCCLH_0015_01_LibTube95_R00250S8A2M0000P0000_C1_KHWGSH_A00090	KUNUSCCLH_0015_01_LibTube75_R00250S8A2M0000P0000_T5_KHWGSH_A00089	0.998571	1	0.9948	0.9949
KUNUSCCLH_0016_01_LibTube96_R00250S8A2M0000P0000_C1_KHWGSH_A00096	KUNUSCCLH_0016_01_LibTube41_R00250S8A2M0000P0000_T1_KHWGSH_A00091	0.999464	1	0.9969	0.9985
KUNUSCCLH_0016_01_LibTube96_R00250S8A2M0000P0000_C1_KHWGSH_A00096	KUNUSCCLH_0016_01_LibTube45_R00250S8A2M0000P0000_T2_KHWGSH_A00092	0.999643	1	0.9979	0.999
KUNUSCCLH_0016_01_LibTube96_R00250S8A2M0000P0000_C1_KHWGSH_A00096	KUNUSCCLH_0016_01_LibTube61_R00250S8A2M0000P0000_T3_KHWGSH_A00093	0.999643	1	0.9979	0.999
KUNUSCCLH_0016_01_LibTube96_R00250S8A2M0000P0000_C1_KHWGSH_A00096	KUNUSCCLH_0016_01_LibTube69_R00250S8A2M0000P0000_T4_KHWGSH_A00094	0.999821	1	0.999	0.9995
KUNUSCCLH_0016_01_LibTube96_R00250S8A2M0000P0000_C1_KHWGSH_A00096	KUNUSCCLH_0016_01_LibTube76_R00250S8A2M0000P0000_T5_KHWGSH_A00095	0.999821	1	0.999	0.9995

Table S3 SNPsniffer analysis to calcualte genotype concordnace between crypt vs bulk.

BAM1	BAM2	Shared _Calls	Matching _Calls	Ratio
KUNUSCCLH_0001_01_LibTube81_R00250S8A2M0000P0000_C1_KHWGSH_A00006	KUNUSCCLH_0001_01_LibTube01_R00250S8A2M0000P0000_T1_KHWGSH_A00001	297	297	1
KUNUSCCLH_0001_01_LibTube81_R00250S8A2M0000P0000_C1_KHWGSH_A00006	KUNUSCCLH_0001_01_LibTube11_R00250S8A2M0000P0000_T2_KHWGSH_A00002	297	297	1
KUNUSCCLH_0001_01_LibTube81_R00250S8A2M0000P0000_C1_KHWGSH_A00006	KUNUSCCLH_0001_01_LibTube19_R00250S8A2M0000P0000_T3_KHWGSH_A00003	297	297	1
KUNUSCCLH_0001_01_LibTube81_R00250S8A2M0000P0000_C1_KHWGSH_A00006	KUNUSCCLH_0001_01_LibTube70_R00250S8A2M0000P0000_T4_KHWGSH_A00004	297	297	1
KUNUSCCLH_0001_01_LibTube81_R00250S8A2M0000P0000_C1_KHWGSH_A00006	KUNUSCCLH_0001_01_LibTube77_R00250S8A2M0000P0000_T5_KHWGSH_A00005	297	297	1
KUNUSCCLH_0002_01_LibTube82_R00250S8A2M0000P0000_C1_KHWGSH_A00012	KUNUSCCLH_0002_01_LibTube12_R00250S8A2M0000P0000_T3_KHWGSH_A00009	276	275	0.996
KUNUSCCLH_0002_01_LibTube82_R00250S8A2M0000P0000_C1_KHWGSH_A00012	KUNUSCCLH_0002_01_LibTube2_R00250S8A2M0000P0000_T1_KHWGSH_A00007	276	276	1
KUNUSCCLH_0002_01_LibTube82_R00250S8A2M0000P0000_C1_KHWGSH_A00012	KUNUSCCLH_0002_01_LibTube26_R00250S8A2M0000P0000_T4_KHWGSH_A00010	276	276	1
KUNUSCCLH_0002_01_LibTube82_R00250S8A2M0000P0000_C1_KHWGSH_A00012	KUNUSCCLH_0002_01_LibTube5_R00250S8A2M0000P0000_T2_KHWGSH_A00008	275	275	1
KUNUSCCLH_0002_01_LibTube82_R00250S8A2M0000P0000_C1_KHWGSH_A00012	KUNUSCCLH_0002_01_LibTube71_R00250S8A2M0000P0000_T5_KHWGSH_A00011	276	276	1
KUNUSCCLH_0003_01_LibTube83_R00250S8A2M0000P0000_C1_KHWGSH_A00018	KUNUSCCLH_0003_01_LibTube13_R00250S8A2M0000P0000_T2_KHWGSH_A00014	283	280	0.989
KUNUSCCLH_0003_01_LibTube83_R00250S8A2M0000P0000_C1_KHWGSH_A00018	KUNUSCCLH_0003_01_LibTube20_R00250S8A2M0000P0000_T3_KHWGSH_A00015	283	283	1
KUNUSCCLH_0003_01_LibTube83_R00250S8A2M0000P0000_C1_KHWGSH_A00018	KUNUSCCLH_0003_01_LibTube27_R00250S8A2M0000P0000_T4_KHWGSH_A00016	283	283	1
KUNUSCCLH_0003_01_LibTube83_R00250S8A2M0000P0000_C1_KHWGSH_A00018	KUNUSCCLH_0003_01_LibTube28_R00250S8A2M0000P0000_T5_KHWGSH_A00017	283	283	1
KUNUSCCLH_0003_01_LibTube83_R00250S8A2M0000P0000_C1_KHWGSH_A00018	KUNUSCCLH_0003_01_LibTube6_R00250S8A2M0000P0000_T1_KHWGSH_A00013	283	282	0.996
KUNUSCCLH_0004_01_LibTube84_R00250S8A2M0000P0000_C1_KHWGSH_A00024	KUNUSCCLH_0004_01_LibTube07_R00250S8A2M0000P0000_T1_KHWGSH_A00019	282	282	1
KUNUSCCLH_0004_01_LibTube84_R00250S8A2M0000P0000_C1_KHWGSH_A00024	KUNUSCCLH_0004_01_LibTube14_R00250S8A2M0000P0000_T2_KHWGSH_A00020	282	282	1
KUNUSCCLH_0004_01_LibTube84_R00250S8A2M0000P0000_C1_KHWGSH_A00024	KUNUSCCLH_0004_01_LibTube21_R00250S8A2M0000P0000_T3_KHWGSH_A00021	282	282	1
KUNUSCCLH_0004_01_LibTube84_R00250S8A2M0000P0000_C1_KHWGSH_A00024	KUNUSCCLH_0004_01_LibTube29_R00250S8A2M0000P0000_T4_KHWGSH_A00022	282	282	1
KUNUSCCLH_0004_01_LibTube84_R00250S8A2M0000P0000_C1_KHWGSH_A00024	KUNUSCCLH_0004_01_LibTube72_R00250S8A2M0000P0000_T5_KHWGSH_A00023	282	282	1
KUNUSCCLH_0005_01_LibTube85_R00250S8A2M0000P0000_C1_KHWGSH_A00030	KUNUSCCLH_0005_01_LibTube08_R00250S8A2M0000P0000_T1_KHWGSH_A00025	291	291	1
KUNUSCCLH_0005_01_LibTube85_R00250S8A2M0000P0000_C1_KHWGSH_A00030	KUNUSCCLH_0005_01_LibTube15_R00250S8A2M0000P0000_T2_KHWGSH_A00026	291	291	1
KUNUSCCLH_0005_01_LibTube85_R00250S8A2M0000P0000_C1_KHWGSH_A00030	KUNUSCCLH_0005_01_LibTube22_R00250S8A2M0000P0000_T3_KHWGSH_A00027	291	291	1
KUNUSCCLH_0005_01_LibTube85_R00250S8A2M0000P0000_C1_KHWGSH_A00030	KUNUSCCLH_0005_01_LibTube30_R00250S8A2M0000P0000_T4_KHWGSH_A00028	291	291	1
KUNUSCCLH_0005_01_LibTube85_R00250S8A2M0000P0000_C1_KHWGSH_A00030	KUNUSCCLH_0005_01_LibTube42_R00250S8A2M0000P0000_T5_KHWGSH_A00029	291	291	1
KUNUSCCLH_0006_01_LibTube86_R00250S8A2M0000P0000_C1_KHWGSH_A00036	KUNUSCCLH_0006_01_LibTube16_R00250S8A2M0000P0000_T1_KHWGSH_A00031	285	285	1
KUNUSCCLH_0006_01_LibTube86_R00250S8A2M0000P0000_C1_KHWGSH_A00036	KUNUSCCLH_0006_01_LibTube23_R00250S8A2M0000P0000_T2_KHWGSH_A00032	285	285	1
KUNUSCCLH_0006_01_LibTube86_R00250S8A2M0000P0000_C1_KHWGSH_A00036	KUNUSCCLH_0006_01_LibTube31_R00250S8A2M0000P0000_T3_KHWGSH_A00033	285	285	1
KUNUSCCLH_0006_01_LibTube86_R00250S8A2M0000P0000_C1_KHWGSH_A00036	KUNUSCCLH_0006_01_LibTube43_R00250S8A2M0000P0000_T4_KHWGSH_A00034	285	285	1
KUNUSCCLH_0006_01_LibTube86_R00250S8A2M0000P0000_C1_KHWGSH_A00036	KUNUSCCLH_0006_01_LibTube78_R00250S8A2M0000P0000_T5_KHWGSH_A00035	285	285	1
KUNUSCCLH_0007_01_LibTube87_R00250S8A2M0000P0000_C1_KHWGSH_A00042	KUNUSCCLH_0007_01_LibTube17_R00250S8A2M0000P0000_T2_KHWGSH_A00038	287	286	0.996
KUNUSCCLH_0007_01_LibTube87_R00250S8A2M0000P0000_C1_KHWGSH_A00042	KUNUSCCLH_0007_01_LibTube24_R00250S8A2M0000P0000_T3_KHWGSH_A00039	287	287	1
KUNUSCCLH_0007_01_LibTube87_R00250S8A2M0000P0000_C1_KHWGSH_A00042	KUNUSCCLH_0007_01_LibTube32_R00250S8A2M0000P0000_T4_KHWGSH_A00040	287	287	1
KUNUSCCLH_0007_01_LibTube87_R00250S8A2M0000P0000_C1_KHWGSH_A00042	KUNUSCCLH_0007_01_LibTube55_R00250S8A2M0000P0000_T5_KHWGSH_A00041	287	287	1
KUNUSCCLH_0007_01_LibTube87_R00250S8A2M0000P0000_C1_KHWGSH_A00042	KUNUSCCLH_0007_01_LibTube9_R00250S8A2M0000P0000_T1_KHWGSH_A00037	287	287	1
KUNUSCCLH_0008_01_LibTube88_R00250S8A2M0000P0000_C1_KHWGSH_A00048	KUNUSCCLH_0008_01_LibTube10_R00250S8A2M0000P0000_T1_KHWGSH_A00043	288	288	1
KUNUSCCLH_0008_01_LibTube88_R00250S8A2M0000P0000_C1_KHWGSH_A00048	KUNUSCCLH_0008_01_LibTube18_R00250S8A2M0000P0000_T2_KHWGSH_A00044	288	288	1
KUNUSCCLH_0008_01_LibTube88_R00250S8A2M0000P0000_C1_KHWGSH_A00048	KUNUSCCLH_0008_01_LibTube25_R00250S8A2M0000P0000_T3_KHWGSH_A00045	288	288	1
KUNUSCCLH_0008_01_LibTube88_R00250S8A2M0000P0000_C1_KHWGSH_A00048	KUNUSCCLH_0008_01_LibTube33_R00250S8A2M0000P0000_T4_KHWGSH_A00046	288	288	1
KUNUSCCLH_0008_01_LibTube88_R00250S8A2M0000P0000_C1_KHWGSH_A00048	KUNUSCCLH_0008_01_LibTube62_R00250S8A2M0000P0000_T5_KHWGSH_A00047	288	288	1
KUNUSCCLH_0009_01_LibTube89_R00250S8A2M0000P0000_C1_KHWGSH_A00054	KUNUSCCLH_0009_01_LibTube34_R00250S8A2M0000P0000_T1_KHWGSH_A00049	292	292	1
KUNUSCCLH_0009_01_LibTube89_R00250S8A2M0000P0000_C1_KHWGSH_A00054	KUNUSCCLH_0009_01_LibTube46_R00250S8A2M0000P0000_T2_KHWGSH_A00050	292	292	1
KUNUSCCLH_0009_01_LibTube89_R00250S8A2M0000P0000_C1_KHWGSH_A00054	KUNUSCCLH_0009_01_LibTube56_R00250S8A2M0000P0000_T3_KHWGSH_A00051	291	291	1
KUNUSCCLH_0009_01_LibTube89_R00250S8A2M0000P0000_C1_KHWGSH_A00054	KUNUSCCLH_0009_01_LibTube63_R00250S8A2M0000P0000_T4_KHWGSH_A00052	292	292	1
KUNUSCCLH_0009_01_LibTube89_R00250S8A2M0000P0000_C1_KHWGSH_A00054	KUNUSCCLH_0009_01_LibTube73_R00250S8A2M0000P0000_T5_KHWGSH_A00053	292	292	1
KUNUSCCLH_0010_01_LibTube90_R00250S8A2M0000P0000_C1_KHWGSH_A00060	KUNUSCCLH_0010_01_LibTube35_R00250S8A2M0000P0000_T1_KHWGSH_A00055	262	262	1
KUNUSCCLH_0010_01_LibTube90_R00250S8A2M0000P0000_C1_KHWGSH_A00060	KUNUSCCLH_0010_01_LibTube47_R00250S8A2M0000P0000_T2_KHWGSH_A00056	262	262	1
KUNUSCCLH_0010_01_LibTube90_R00250S8A2M0000P0000_C1_KHWGSH_A00060	KUNUSCCLH_0010_01_LibTube64_R00250S8A2M0000P0000_T3_KHWGSH_A00057	262	262	1
KUNUSCCLH_0010_01_LibTube90_R00250S8A2M0000P0000_C1_KHWGSH_A00060	KUNUSCCLH_0010_01_LibTube79_R00250S8A2M0000P0000_T4_KHWGSH_A00058	262	262	1
KUNUSCCLH_0010_01_LibTube90_R00250S8A2M0000P0000_C1_KHWGSH_A00060	KUNUSCCLH_0010_01_LibTube80_R00250S8A2M0000P0000_T5_KHWGSH_A00059	262	262	1
KUNUSCCLH_0011_01_LibTube91_R00250S8A2M0000P0000_C1_KHWGSH_A00066	KUNUSCCLH_0011_01_LibTube36_R00250S8A2M0000P0000_T1_KHWGSH_A00061	267	266	0.996
KUNUSCCLH_0011_01_LibTube91_R00250S8A2M0000P0000_C1_KHWGSH_A00066	KUNUSCCLH_0011_01_LibTube48_R00250S8A2M0000P0000_T2_KHWGSH_A00062	267	267	1
KUNUSCCLH_0011_01_LibTube91_R00250S8A2M0000P0000_C1_KHWGSH_A00066	KUNUSCCLH_0011_01_LibTube52_R00250S8A2M0000P0000_T3_KHWGSH_A00063	267	267	1
KUNUSCCLH_0011_01_LibTube91_R00250S8A2M0000P0000_C1_KHWGSH_A00066	KUNUSCCLH_0011_01_LibTube57_R00250S8A2M0000P0000_T4_KHWGSH_A00064	267	267	1
KUNUSCCLH_0011_01_LibTube91_R00250S8A2M0000P0000_C1_KHWGSH_A00066	KUNUSCCLH_0011_01_LibTube65_R00250S8A2M0000P0000_T5_KHWGSH_A00065	267	267	1
KUNUSCCLH_0012_01_LibTube92_R00250S8A2M0000P0000_C1_KHWGSH_A00072	KUNUSCCLH_0012_01_LibTube3_R00250S8A2M0000P0000_T1_KHWGSH_A00067	275	275	1
KUNUSCCLH_0012_01_LibTube92_R00250S8A2M0000P0000_C1_KHWGSH_A00072	KUNUSCCLH_0012_01_LibTube37_R00250S8A2M0000P0000_T3_KHWGSH_A00069	275	275	1
KUNUSCCLH_0012_01_LibTube92_R00250S8A2M0000P0000_C1_KHWGSH_A00072	KUNUSCCLH_0012_01_LibTube49_R00250S8A2M0000P0000_T4_KHWGSH_A00070	275	275	1
KUNUSCCLH_0012_01_LibTube92_R00250S8A2M0000P0000_C1_KHWGSH_A00072	KUNUSCCLH_0012_01_LibTube53_R00250S8A2M0000P0000_T5_KHWGSH_A00071	275	275	1
KUNUSCCLH_0012_01_LibTube92_R00250S8A2M0000P0000_C1_KHWGSH_A00072	KUNUSCCLH_0012_01_LibTube98_R00250S8A2M0000P0000_T6_KHWGSH_A00097	275	275	1
KUNUSCCLH_0012_01_LibTube92_R00250S8A2M0000P0000_C1_KHWGSH_A00072	KUNUSCCLH_0012_01_LibTube99_R00250S8A2M0000P0000_T7_KHWGSH_A00098	275	275	1
KUNUSCCLH_0013_01_LibTube93_R00250S8A2M0000P0000_C1_KHWGSH_A00078	KUNUSCCLH_0013_01_LibTube38_R00250S8A2M0000P0000_T1_KHWGSH_A00073	273	273	1
KUNUSCCLH_0013_01_LibTube93_R00250S8A2M0000P0000_C1_KHWGSH_A00078	KUNUSCCLH_0013_01_LibTube50_R00250S8A2M0000P0000_T2_KHWGSH_A00074	273	273	1
KUNUSCCLH_0013_01_LibTube93_R00250S8A2M0000P0000_C1_KHWGSH_A00078	KUNUSCCLH_0013_01_LibTube58_R00250S8A2M0000P0000_T3_KHWGSH_A00075	273	273	1
KUNUSCCLH_0013_01_LibTube93_R00250S8A2M0000P0000_C1_KHWGSH_A00078	KUNUSCCLH_0013_01_LibTube66_R00250S8A2M0000P0000_T4_KHWGSH_A00076	273	273	1
KUNUSCCLH_0013_01_LibTube93_R00250S8A2M0000P0000_C1_KHWGSH_A00078	KUNUSCCLH_0013_01_LibTube74_R00250S8A2M0000P0000_T5_KHWGSH_A00077	273	273	1
KUNUSCCLH_0014_01_LibTube94_R00250S8A2M0000P0000_C1_KHWGSH_A00084	KUNUSCCLH_0014_01_LibTube39_R00250S8A2M0000P0000_T1_KHWGSH_A00079	282	282	1
KUNUSCCLH_0014_01_LibTube94_R00250S8A2M0000P0000_C1_KHWGSH_A00084	KUNUSCCLH_0014_01_LibTube51_R00250S8A2M0000P0000_T2_KHWGSH_A00080	282	282	1
KUNUSCCLH_0014_01_LibTube94_R00250S8A2M0000P0000_C1_KHWGSH_A00084	KUNUSCCLH_0014_01_LibTube54_R00250S8A2M0000P0000_T3_KHWGSH_A00081	282	282	1
KUNUSCCLH_0014_01_LibTube94_R00250S8A2M0000P0000_C1_KHWGSH_A00084	KUNUSCCLH_0014_01_LibTube59_R00250S8A2M0000P0000_T4_KHWGSH_A00082	282	282	1
KUNUSCCLH_0014_01_LibTube94_R00250S8A2M0000P0000_C1_KHWGSH_A00084	KUNUSCCLH_0014_01_LibTube67_R00250S8A2M0000P0000_T5_KHWGSH_A00083	282	282	1
KUNUSCCLH_0015_01_LibTube95_R00250S8A2M0000P0000_C1_KHWGSH_A00090	KUNUSCCLH_0015_01_LibTube40_R00250S8A2M0000P0000_T1_KHWGSH_A00085	270	270	1
KUNUSCCLH_0015_01_LibTube95_R00250S8A2M0000P0000_C1_KHWGSH_A00090	KUNUSCCLH_0015_01_LibTube44_R00250S8A2M0000P0000_T2_KHWGSH_A00086	270	268	0.992
KUNUSCCLH_0015_01_LibTube95_R00250S8A2M0000P0000_C1_KHWGSH_A00090	KUNUSCCLH_0015_01_LibTube60_R00250S8A2M0000P0000_T3_KHWGSH_A00087	270	270	1
KUNUSCCLH_0015_01_LibTube95_R00250S8A2M0000P0000_C1_KHWGSH_A00090	KUNUSCCLH_0015_01_LibTube68_R00250S8A2M0000P0000_T4_KHWGSH_A00088	270	270	1
KUNUSCCLH_0015_01_LibTube95_R00250S8A2M0000P0000_C1_KHWGSH_A00090	KUNUSCCLH_0015_01_LibTube75_R00250S8A2M0000P0000_T5_KHWGSH_A00089	270	270	1
KUNUSCCLH_0016_01_LibTube96_R00250S8A2M0000P0000_C1_KHWGSH_A00096	KUNUSCCLH_0016_01_LibTube41_R00250S8A2M0000P0000_T1_KHWGSH_A00091	277	276	0.996
KUNUSCCLH_0016_01_LibTube96_R00250S8A2M0000P0000_C1_KHWGSH_A00096	KUNUSCCLH_0016_01_LibTube45_R00250S8A2M0000P0000_T2_KHWGSH_A00092	277	276	0.996
KUNUSCCLH_0016_01_LibTube96_R00250S8A2M0000P0000_C1_KHWGSH_A00096	KUNUSCCLH_0016_01_LibTube61_R00250S8A2M0000P0000_T3_KHWGSH_A00093	277	276	0.996
KUNUSCCLH_0016_01_LibTube96_R00250S8A2M0000P0000_C1_KHWGSH_A00096	KUNUSCCLH_0016_01_LibTube69_R00250S8A2M0000P0000_T4_KHWGSH_A00094	276	275	0.996
KUNUSCCLH_0016_01_LibTube96_R00250S8A2M0000P0000_C1_KHWGSH_A00096	KUNUSCCLH_0016_01_LibTube76_R00250S8A2M0000P0000_T5_KHWGSH_A00095	277	275	0.992

Table S4 Population-based principal component analysis of crypt vs bulk.

SampleID	Sample Type	PC1	PC2	PC3
KUNUSCCLH_0001_01_LibTube01_R00250S8A2M0000P0000_T1_KHWGSH_A00001	Crypt	-56.29752	-10.55852	-7.55690
KUNUSCCLH_0001_01_LibTube11_R00250S8A2M0000P0000_T2_KHWGSH_A00002	Crypt	-56.31777	-10.54544	-7.49554
KUNUSCCLH_0001_01_LibTube19_R00250S8A2M0000P0000_T3_KHWGSH_A00003	Crypt	-56.35430	-10.57444	-7.41230
KUNUSCCLH_0001_01_LibTube70_R00250S8A2M0000P0000_T4_KHWGSH_A00004	Crypt	-56.30202	-10.58060	-7.47768
KUNUSCCLH_0001_01_LibTube77_R00250S8A2M0000P0000_T5_KHWGSH_A00005	Crypt	-56.32585	-10.59753	-7.52504
KUNUSCCLH_0001_01_LibTube81_R00250S8A2M0000P0000_C1_KHWGSH_A00006	Bulk	-56.31880	-10.57569	-7.51747
KUNUSCCLH_0002_01_LibTube12_R00250S8A2M0000P0000_T3_KHWGSH_A00009	Crypt	-54.25466	-18.52475	-3.52116
KUNUSCCLH_0002_01_LibTube26_R00250S8A2M0000P0000_T4_KHWGSH_A00010	Crypt	-54.22159	-18.56108	-3.53539
KUNUSCCLH_0002_01_LibTube2_R00250S8A2M0000P0000_T1_KHWGSH_A00007	Crypt	-54.25152	-18.56788	-3.53366
KUNUSCCLH_0002_01_LibTube5_R00250S8A2M0000P0000_T2_KHWGSH_A00008	Crypt	-54.24189	-18.52454	-3.54831
KUNUSCCLH_0002_01_LibTube71_R00250S8A2M0000P0000_T5_KHWGSH_A00011	Crypt	-54.30537	-18.58241	-3.52506
KUNUSCCLH_0002_01_LibTube82_R00250S8A2M0000P0000_C1_KHWGSH_A00012	Bulk	-54.22906	-18.53449	-3.52308
KUNUSCCLH_0003_01_LibTube13_R00250S8A2M0000P0000_T2_KHWGSH_A00014	Crypt	-64.96521	-18.18374	-11.37783
KUNUSCCLH_0003_01_LibTube20_R00250S8A2M0000P0000_T3_KHWGSH_A00015	Crypt	-64.97800	-18.18941	-11.32948
KUNUSCCLH_0003_01_LibTube27_R00250S8A2M0000P0000_T4_KHWGSH_A00016	Crypt	-64.90050	-18.12944	-11.34767
KUNUSCCLH_0003_01_LibTube28_R00250S8A2M0000P0000_T5_KHWGSH_A00017	Crypt	-64.96009	-18.22159	-11.27624
KUNUSCCLH_0003_01_LibTube6_R00250S8A2M0000P0000_T1_KHWGSH_A00013	Crypt	-64.92316	-18.18681	-11.35875
KUNUSCCLH_0003_01_LibTube83_R00250S8A2M0000P0000_C1_KHWGSH_A00018	Bulk	-64.92490	-18.19135	-11.32142
KUNUSCCLH_0004_01_LibTube07_R00250S8A2M0000P0000_T1_KHWGSH_A00019	Crypt	-62.03934	-24.22313	-5.09084
KUNUSCCLH_0004_01_LibTube14_R00250S8A2M0000P0000_T2_KHWGSH_A00020	Crypt	-62.06585	-24.26999	-5.08497
KUNUSCCLH_0004_01_LibTube21_R00250S8A2M0000P0000_T3_KHWGSH_A00021	Crypt	-62.06556	-24.28022	-5.14235
KUNUSCCLH_0004_01_LibTube29_R00250S8A2M0000P0000_T4_KHWGSH_A00022	Crypt	-61.91150	-23.92279	-5.07948
KUNUSCCLH_0004_01_LibTube72_R00250S8A2M0000P0000_T5_KHWGSH_A00023	Crypt	-62.09424	-24.26408	-5.09019
KUNUSCCLH_0004_01_LibTube84_R00250S8A2M0000P0000_C1_KHWGSH_A00024	Bulk	-62.01611	-24.21641	-5.11203
KUNUSCCLH_0005_01_LibTube08_R00250S8A2M0000P0000_T1_KHWGSH_A00025	Crypt	-63.21500	-19.68724	-10.81719
KUNUSCCLH_0005_01_LibTube15_R00250S8A2M0000P0000_T2_KHWGSH_A00026	Crypt	-63.48276	-19.97217	-10.77986
KUNUSCCLH_0005_01_LibTube22_R00250S8A2M0000P0000_T3_KHWGSH_A00027	Crypt	-63.44492	-20.04104	-10.70697
KUNUSCCLH_0005_01_LibTube30_R00250S8A2M0000P0000_T4_KHWGSH_A00028	Crypt	-63.43338	-20.04614	-10.77006
KUNUSCCLH_0005_01_LibTube42_R00250S8A2M0000P0000_T5_KHWGSH_A00029	Crypt	-63.52114	-20.06273	-10.72425
KUNUSCCLH_0005_01_LibTube85_R00250S8A2M0000P0000_C1_KHWGSH_A00030	Bulk	-63.48004	-20.07118	-10.73213
KUNUSCCLH_0006_01_LibTube16_R00250S8A2M0000P0000_T1_KHWGSH_A00031	Crypt	-59.36943	-20.45983	-6.98820
KUNUSCCLH_0006_01_LibTube23_R00250S8A2M0000P0000_T2_KHWGSH_A00032	Crypt	-59.43462	-20.45837	-7.00995
KUNUSCCLH_0006_01_LibTube31_R00250S8A2M0000P0000_T3_KHWGSH_A00033	Crypt	-59.40872	-20.48823	-7.02232
KUNUSCCLH_0006_01_LibTube43_R00250S8A2M0000P0000_T4_KHWGSH_A00034	Crypt	-59.42295	-20.48485	-7.01333
KUNUSCCLH_0006_01_LibTube78_R00250S8A2M0000P0000_T5_KHWGSH_A00035	Crypt	-59.35620	-20.49683	-7.01636
KUNUSCCLH_0006_01_LibTube86_R00250S8A2M0000P0000_C1_KHWGSH_A00036	Bulk	-59.40793	-20.47136	-6.98460
KUNUSCCLH_0007_01_LibTube9_R00250S8A2M0000P0000_T1_KHWGSH_A00037	Crypt	-63.15699	-22.32388	-3.33545
KUNUSCCLH_0007_01_LibTube17_R00250S8A2M0000P0000_T2_KHWGSH_A00038	Crypt	-63.15701	-22.35575	-3.38428
KUNUSCCLH_0007_01_LibTube24_R00250S8A2M0000P0000_T3_KHWGSH_A00039	Crypt	-63.21880	-22.35069	-3.31396
KUNUSCCLH_0007_01_LibTube32_R00250S8A2M0000P0000_T4_KHWGSH_A00040	Crypt	-63.16642	-22.36022	-3.32509
KUNUSCCLH_0007_01_LibTube55_R00250S8A2M0000P0000_T5_KHWGSH_A00041	Crypt	-63.18078	-22.36614	-3.31939
KUNUSCCLH_0007_01_LibTube87_R00250S8A2M0000P0000_C1_KHWGSH_A00042	Bulk	-63.21717	-22.34470	-3.35251
KUNUSCCLH_0008_01_LibTube10_R00250S8A2M0000P0000_T1_KHWGSH_A00043	Crypt	-58.60257	-19.13898	-9.99616
KUNUSCCLH_0008_01_LibTube18_R00250S8A2M0000P0000_T2_KHWGSH_A00044	Crypt	-58.58168	-19.07339	-9.98650
KUNUSCCLH_0008_01_LibTube25_R00250S8A2M0000P0000_T3_KHWGSH_A00045	Crypt	-58.62590	-19.08831	-9.97345
KUNUSCCLH_0008_01_LibTube33_R00250S8A2M0000P0000_T4_KHWGSH_A00046	Crypt	-58.60571	-19.06605	-9.97263
KUNUSCCLH_0008_01_LibTube62_R00250S8A2M0000P0000_T5_KHWGSH_A00047	Crypt	-58.59672	-19.09921	-9.96482
KUNUSCCLH_0008_01_LibTube88_R00250S8A2M0000P0000_C1_KHWGSH_A00048	Bulk	-58.56305	-19.07978	-10.01873
KUNUSCCLH_0009_01_LibTube34_R00250S8A2M0000P0000_T1_KHWGSH_A00049	Crypt	-55.04364	-16.08283	-14.81769
KUNUSCCLH_0009_01_LibTube46_R00250S8A2M0000P0000_T2_KHWGSH_A00050	Crypt	-54.92753	-15.99527	-14.82274
KUNUSCCLH_0009_01_LibTube56_R00250S8A2M0000P0000_T3_KHWGSH_A00051	Crypt	-54.99100	-15.93871	-14.82583
KUNUSCCLH_0009_01_LibTube63_R00250S8A2M0000P0000_T4_KHWGSH_A00052	Crypt	-54.93791	-15.97927	-14.83600
KUNUSCCLH_0009_01_LibTube73_R00250S8A2M0000P0000_T5_KHWGSH_A00053	Crypt	-54.95064	-15.99116	-14.80402
KUNUSCCLH_0009_01_LibTube89_R00250S8A2M0000P0000_C1_KHWGSH_A00054	Bulk	-55.02470	-16.03176	-14.76079
KUNUSCCLH_0010_01_LibTube35_R00250S8A2M0000P0000_T1_KHWGSH_A00055	Crypt	-62.83368	-21.94723	-5.23711
KUNUSCCLH_0010_01_LibTube47_R00250S8A2M0000P0000_T2_KHWGSH_A00056	Crypt	-62.77938	-21.99102	-5.19295
KUNUSCCLH_0010_01_LibTube64_R00250S8A2M0000P0000_T3_KHWGSH_A00057	Crypt	-62.75501	-21.89980	-5.22257
KUNUSCCLH_0010_01_LibTube79_R00250S8A2M0000P0000_T4_KHWGSH_A00058	Crypt	-62.81527	-21.95340	-5.24137
KUNUSCCLH_0010_01_LibTube80_R00250S8A2M0000P0000_T5_KHWGSH_A00059	Crypt	-62.79005	-21.87944	-5.18010
KUNUSCCLH_0010_01_LibTube90_R00250S8A2M0000P0000_C1_KHWGSH_A00060	Bulk	-62.74871	-21.89841	-5.20237
KUNUSCCLH_0011_01_LibTube36_R00250S8A2M0000P0000_T1_KHWGSH_A00061	Crypt	-59.31933	-4.94876	-11.50711
KUNUSCCLH_0011_01_LibTube48_R00250S8A2M0000P0000_T2_KHWGSH_A00062	Crypt	-59.32586	-4.86293	-11.54315
KUNUSCCLH_0011_01_LibTube52_R00250S8A2M0000P0000_T3_KHWGSH_A00063	Crypt	-59.34010	-4.94060	-11.57434
KUNUSCCLH_0011_01_LibTube57_R00250S8A2M0000P0000_T4_KHWGSH_A00064	Crypt	-59.36746	-4.86627	-11.55331
KUNUSCCLH_0011_01_LibTube65_R00250S8A2M0000P0000_T5_KHWGSH_A00065	Crypt	-59.41192	-4.89105	-11.59997
KUNUSCCLH_0011_01_LibTube91_R00250S8A2M0000P0000_C1_KHWGSH_A00066	Bulk	-59.38446	-4.91468	-11.55663
KUNUSCCLH_0012_01_LibTube37_R00250S8A2M0000P0000_T3_KHWGSH_A00069	Crypt	-60.72794	-16.28174	-12.02312
KUNUSCCLH_0012_01_LibTube3_R00250S8A2M0000P0000_T1_KHWGSH_A00067	Crypt	-60.66951	-16.26396	-12.02951
KUNUSCCLH_0012_01_LibTube49_R00250S8A2M0000P0000_T4_KHWGSH_A00070	Crypt	-60.70933	-16.24541	-12.00705
KUNUSCCLH_0012_01_LibTube53_R00250S8A2M0000P0000_T5_KHWGSH_A00071	Crypt	-60.71334	-16.28619	-12.05229
KUNUSCCLH_0012_01_LibTube98_R00250S8A2M0000P0000_T6_KHWGSH_A00097	Crypt	-60.67155	-16.31969	-12.01111
KUNUSCCLH_0012_01_LibTube99_R00250S8A2M0000P0000_T7_KHWGSH_A00098	Crypt	-60.69218	-16.25088	-12.02653
KUNUSCCLH_0012_01_LibTube92_R00250S8A2M0000P0000_C1_KHWGSH_A00072	Bulk	-60.71197	-16.29496	-12.02258
KUNUSCCLH_0013_01_LibTube38_R00250S8A2M0000P0000_T1_KHWGSH_A00073	Crypt	-60.92050	-14.56538	-8.93196
KUNUSCCLH_0013_01_LibTube50_R00250S8A2M0000P0000_T2_KHWGSH_A00074	Crypt	-60.95109	-14.51123	-8.88849
KUNUSCCLH_0013_01_LibTube58_R00250S8A2M0000P0000_T3_KHWGSH_A00075	Crypt	-60.86661	-14.44914	-8.90460
KUNUSCCLH_0013_01_LibTube66_R00250S8A2M0000P0000_T4_KHWGSH_A00076	Crypt	-60.97896	-14.51353	-8.94317
KUNUSCCLH_0013_01_LibTube74_R00250S8A2M0000P0000_T5_KHWGSH_A00077	Crypt	-60.95887	-14.48307	-8.94730
KUNUSCCLH_0013_01_LibTube93_R00250S8A2M0000P0000_C1_KHWGSH_A00078	Bulk	-60.78902	-14.47578	-9.00840
KUNUSCCLH_0014_01_LibTube39_R00250S8A2M0000P0000_T1_KHWGSH_A00079	Crypt	-64.84479	17.17523	-6.50672
KUNUSCCLH_0014_01_LibTube51_R00250S8A2M0000P0000_T2_KHWGSH_A00080	Crypt	-64.83722	17.23138	-6.52555
KUNUSCCLH_0014_01_LibTube54_R00250S8A2M0000P0000_T3_KHWGSH_A00081	Crypt	-64.91245	17.23479	-6.51670
KUNUSCCLH_0014_01_LibTube59_R00250S8A2M0000P0000_T4_KHWGSH_A00082	Crypt	-64.86753	17.19853	-6.48966
KUNUSCCLH_0014_01_LibTube67_R00250S8A2M0000P0000_T5_KHWGSH_A00083	Crypt	-64.82951	17.28488	-6.52739
KUNUSCCLH_0014_01_LibTube94_R00250S8A2M0000P0000_C1_KHWGSH_A00084	Bulk	-64.59359	17.29044	-6.53240
KUNUSCCLH_0015_01_LibTube40_R00250S8A2M0000P0000_T1_KHWGSH_A00085	Crypt	-71.90836	-1.50870	-14.17252
KUNUSCCLH_0015_01_LibTube44_R00250S8A2M0000P0000_T2_KHWGSH_A00086	Crypt	-71.78067	-1.51245	-14.29218
KUNUSCCLH_0015_01_LibTube60_R00250S8A2M0000P0000_T3_KHWGSH_A00087	Crypt	-71.91135	-1.51507	-14.20557
KUNUSCCLH_0015_01_LibTube68_R00250S8A2M0000P0000_T4_KHWGSH_A00088	Crypt	-71.78858	-1.44006	-14.32027
KUNUSCCLH_0015_01_LibTube75_R00250S8A2M0000P0000_T5_KHWGSH_A00089	Crypt	-71.89845	-1.51053	-14.26915
KUNUSCCLH_0015_01_LibTube95_R00250S8A2M0000P0000_C1_KHWGSH_A00090	Bulk	-71.70359	-1.45052	-14.31873
KUNUSCCLH_0016_01_LibTube41_R00250S8A2M0000P0000_T1_KHWGSH_A00091	Crypt	-64.65142	10.52669	-5.60516
KUNUSCCLH_0016_01_LibTube45_R00250S8A2M0000P0000_T2_KHWGSH_A00092	Crypt	-64.57528	10.58763	-5.69322
KUNUSCCLH_0016_01_LibTube61_R00250S8A2M0000P0000_T3_KHWGSH_A00093	Crypt	-64.64276	10.62040	-5.68650
KUNUSCCLH_0016_01_LibTube69_R00250S8A2M0000P0000_T4_KHWGSH_A00094	Crypt	-64.59464	10.58661	-5.68672
KUNUSCCLH_0016_01_LibTube76_R00250S8A2M0000P0000_T5_KHWGSH_A00095	Crypt	-64.67225	10.56988	-5.65883
KUNUSCCLH_0016_01_LibTube96_R00250S8A2M0000P0000_C1_KHWGSH_A00096	Bulk	-64.49662	10.65095	-5.60824
